# Elucidation of the Translation Initiation Factor Interaction Network of *Haloferax volcanii* Reveals Coupling of Transcription and Translation in Haloarchaea

**DOI:** 10.3389/fmicb.2021.742806

**Published:** 2021-10-26

**Authors:** Franziska Schramm, Andreas Borst, Uwe Linne, Jörg Soppa

**Affiliations:** ^1^Institute for Molecular Biosciences, Biocentre, Goethe-University, Frankfurt, Germany; ^2^Mass Spectrometry Facility, Department of Chemistry, Phillipps University Marburg, Marburg, Germany

**Keywords:** *Haloferax volcanii*, translation initiation, aIF, ribosome, RNA polymerase, interaction networks, transcription, coupling

## Abstract

Translation is an important step in gene expression. Initiation of translation is rate-limiting, and it is phylogenetically more diverse than elongation or termination. Bacteria contain only three initiation factors. In stark contrast, eukaryotes contain more than 10 (subunits of) initiation factors (eIFs). The genomes of archaea contain many genes that are annotated to encode archaeal homologs of eukaryotic initiation factors (aIFs). However, experimental characterization of aIFs is scarce and mostly restricted to very few species. To broaden the view, the protein–protein interaction network of aIFs in the halophilic archaeon *Haloferax volcanii* has been characterized. To this end, tagged versions of 14 aIFs were overproduced, affinity isolated, and the co-isolated binding partners were identified by peptide mass fingerprinting and MS/MS analyses. The aIF–aIF interaction network was resolved, and it was found to contain two interaction hubs, (1) the universally conserved factor aIF5B, and (2) a protein that has been annotated as the enzyme ribose-1,5-bisphosphate isomerase, which we propose to rename to aIF2Bα. Affinity isolation of aIFs also led to the co-isolation of many ribosomal proteins, but also transcription factors and subunits of the RNA polymerase (Rpo). To analyze a possible coupling of transcription and translation, seven tagged Rpo subunits were overproduced, affinity isolated, and co-isolated proteins were identified. The Rpo interaction network contained many transcription factors, but also many ribosomal proteins as well as the initiation factors aIF5B and aIF2Bα. These results showed that transcription and translation are coupled in haloarchaea, like in *Escherichia coli*. It seems that aIF5B and aIF2Bα are not only interaction hubs in the translation initiation network, but also key players in the transcription-translation coupling.

## Introduction

Translation is a very important step in the process of expression of the genome information into the phenotype of cells and organisms. Translation is evolutionary very old, and ribosomes were already present in the Last Universal Common Ancestor of all living beings from the three domains of life (Fox, 2010; Opron and Burton, 2018; Bowman et al., 2020). In fact, comparison of the 16S/18S rRNA has led to the proposal that a third domain of life exist, the archaea, which are not closely related to the second group of prokaryotes, the bacteria (Woese and Fox, 1977). Initially this was based on very few species of methanogenic archaea, however, the molecular distinction between archaea and bacteria based on rRNA sequences has held true after the isolation of hundreds of new species and thousands of rRNA sequences generated by metagenomics. Recently, the three domain concept of life has been challenged, but this does not concern the dichotomy of archaea and bacteria. Instead, the recent discovery of many new groups of archaea currently makes it more likely that the eukaryotes evolved from within the archaea, and thus, that only two major primary domains exist (Eme et al., 2017; Liu et al., 2021). While in evolution different phylogenetic groups added additional subdomains into the rRNA sequences and added lineage-specific ribosomal proteins, a structural core of the ribosomal RNA exists that is shared by archaea, bacteria, and eukaryotes, and the majority of ribosomal proteins are universal (Bernier et al., 2018).

Translation is comprised of the steps’ initiation, elongation, termination, and ribosome recycling. Initiation of translation is phylogenetically most diverse among these four steps, and at least five different mechanisms exist. In eukaryotes, canonical translation initiation involves recognition of the 5'-cap structure of mRNAs and scanning of the small 40S ribosomal subunit along the mRNA, until the start codon is reached. Then, the large 60S ribosomal subunit joins, and translation elongation can start. An alternative translation initiation mechanism in eukaryotes involves Internal Ribosome Entry Sites (IRES). These are specific structures within the 5'-UTRs of transcripts (or in intergenic regions of bicistronic viral transcripts) that are recognized by specific proteins, so-called IRES Trans-Acting Factors, which attract the 40S subunit to the internal sites. Various eukaryotic translation initiation factors (eIFs) are involved in and essential for translation initiation (see below). Several reviews summarize different aspects of translation initiation in eukaryotes (Dever et al., 2016; Andreev et al., 2017; Aylett and Ban, 2017; Hinnebusch, 2017; Guca and Hashem, 2018; Shirokikh and Preiss, 2018; Weisser and Ban, 2019).

In bacteria, canonical translation initiation involves base-pairing between the so-called Shine Dalgarno (SD) motif in the mRNA, which is localized a few nucleotides upstream of the start codon, and the anti-SD motif, which is localized at the 3'-end of the 16S rRNA. Thereby, the start codon is localized in the P-site of the small 30S rRNA, and the large 50S subunit can join, before elongation can start. The internal recognition of start sites enables the formation of polycistronic mRNAs, which contain several to many genes. Also in bacteria, initiation factors (IFs) are involved in the process (see below). In addition to the canonical transcripts, also non-canonical transcripts exist in bacteria, which either contain a 5'-UTR lacking a SD motif or lack a 5'-UTR and are leaderless. The fractions of the three groups of transcripts differ widely in different phylogenetic groups of bacteria. For example, the SD mechanism for translation initiation is not functional at all in Bacteriodetes (Accetto and Avguštin, 2011), and the fractions of SD-led genes are rather low in Chlamydia and cyanobacteria (Huber et al., 2019). Translation initiation in bacteria has been reviewed intensively (Marintchev and Wagner, 2004; Kaberdin and Bläsi, 2006; Simonetti et al., 2009; Malys and McCarthy, 2011; Milón and Rodnina, 2012; Duval et al., 2015; Gualerzi and Pon, 2015).

Archaea contain the same three types of transcripts as bacteria, i.e., (1) canonical transcripts with 5'-UTRs and SD motif, (2) non-canonical transcripts with 5'-UTRs lacking an SD motif, and (3) leaderless transcripts. The distribution is very different in various groups of archaea. For example, transcripts in methanogenic archaea typically have very long 5'-UTRs with SD motifs, while, in stark contrast, transcripts in haloarchaea and *Sulfolobales* are typically leaderless. A dRNA-Seq study has shown that 72% of all transcripts of *Haloferax volcanii* are leaderless and the fraction of transcripts with SD motifs is extremely low (Babski et al., 2016). In addition, SD motifs are non-functional for translation initiation at 5'-UTRs in *H. volcanii* (Kramer et al., 2014). Several reviews summarize various aspects about translation initiation in archaea, and compare it with initiation in bacteria and eukaryotes (Londei, 2005; Benelli and Londei, 2009, 2011; Schmitt et al., 2019, 2020).

In stark contrast to the similarities in the classes of transcripts, the numbers of translation initiation factors are totally different in bacteria and archaea. Bacteria contain only three initiation factors, IF1, IF2, and IF3. IF1 is homologous to the archaeal factor aIF1A and the eukaryotic factor eIF1A, and it is thus universally conserved. The second universally conserved factor is IF2, which is homologous to the archaeal factor aIF5B and the eukaryotic factor eIF5B. The third bacterial factor, IF3, has some structural similarities with the factors aIF1 and eIF1, but the sequences and topologies are different, and thus, bacterial IF3 and aIF1/eIF1 are not homologues.

Archaea and eukaryotes share several additional factors, which are not present in bacteria. A central factor is the heterotrimeric factor aIF2/eIF2, which binds the initiator tRNA and brings it into the P-site of the ribosome. aIF1 and eIF1 are homologous and are found in the preinitiation complex of archaea and eukaryotes together with aIF2/eIF2 and aIF1A/eIF1A, thus enhancing the accuracy of start codon selection (Schmitt et al., 2020). The eukaryotic factor eIF4F consists of three subunits, a homolog of one of which, a/eIF4A, is encoded in many archaeal genomes. However, it cannot have the same function as the eukaryotic factor. The eukaryotic factor eIF4F binds to the cap of eukaryotic transcripts and brings the preinitiation complex to the mRNA 5'-end. However, archaeal transcripts do not have a 5'-cap, and thus there is no use for a cap-binding factor. Therefore, it is not clear whether aIF4A has a function in translation initiation at all.

Another initiation factor in eukaryotes is eIF2B. It consists of a catalytic subcomplex of two subunits (eIF2Bγ, eIF2Bε) and a regulatory subunit of three subunits (eIF2Bα, eIF2Bβ, eIF2Bδ). The whole complex is a decamer, because there are two copies of each of the five subunits in the complex (Bogorad et al., 2014; Schoof et al., 2021). eIF2B binds to the central factor eIF2 and catalyzes the exchange of GDP against GTP. The two catalytic subunits are not encoded in archaea, while there are genes for the three regulatory subunits in many archaeal genomes. The biological role of these “regulatory proteins” in the absence of their catalytic binding partners is unknown. It could be shown that a presumed aIF2B subunit from three species, *Pyrococcus horikoshii*, *Pyrococcus furiosus*, and *Thermoplasma acidophilum*, binds to the alpha subunit of aIF2 of the cognate species *in vitro* (Dev et al., 2009), indicating that the aIF2B subunits might have some – as yet unknown – function in archaeal translation initiation.

In recent years considerable progress in the experimental characterization of translation initiation has been obtained, albeit the number of studies is much lower than the number of studies in eukaryotes or in bacteria (in Pubmed the numbers of studies with “translation initiation” AND archae*, bacteri*, or eukaryote* in Title/Abstract are 187, 892, and 4,890, respectively). By far the highest number of studies have been performed with the Crenarchaeon *Sulfolobus solfataricus* (La Teana et al., 2013; Schmitt et al., 2020). Structures of preinitiation complexes have been solved with constituents from *Pyrococcus abyssi* (Coureux et al., 2016, 2020). The number of studies with halophilic archaea is very low. As mentioned above, it could be shown that the SD motif is non-functional for translation initiation *in vivo* (Kramer et al., 2014), and that a novel mechanism for translation initiation operates (Hering et al., 2009). It was also revealed that the 5'-ends and 3'-ends of *H. volcanii* transcripts functionally interact *in vivo* (Brenneis and Soppa, 2009). In a very comprehensive study with 14 genes that were annotated to encode (subunits of) translation initiation factors, all nine non-essential genes were deleted and all five essential genes were conditionally depleted, and the consequences for the phenotype of the mutants were characterized (Gäbel et al., 2013). In the present study we have extended this approach, and 14 proteins with the annotation to be (subunits of) translation initiation factors of *H. volcanii* were overproduced as tagged variants. After affinity purification, co-isolated binding partners were identified by peptide mass fingerprinting and MS–MS analyses. Thereby, the protein–protein interaction network of haloarchaeal translation initiation could be resolved. The unexpected co-isolation of several subunits of the RNA polymerase prompted us to extend the project further. To this end, seven subunits of the RNA polymerase were overproduced, and the RNA polymerase interaction network was also elucidated using co-affinity isolation and MS as well as MS/MS analyses. Together, we report here a very comprehensive analysis of the interaction network of 21 proteins, which was controlled by two very strict negative controls, i.e., cultures containing an empty vector and cultures overproducing a metabolic enzyme.

## Materials and Methods

### Strains, Media and Culture Conditions

The strain *H. volcanii* H26 was obtained from Thorsten Allers (Nottingham, United Kingdom), it is a *pyrE* deletion strain lacking the plasmid pHV2. The deletion of the *dhfr* (dihydrofolate reductase) gene HVO_1279 has been described previously (Maurer et al., 2018). The deletion strains of genes encoding translation initiation factors have been generated in a previous study (Gäbel et al., 2013). Multi cycle PCRs were used to confirm that all mutants were still homozygous. The sequences of oligonucleotides are listed in Supplementary Table S1. In a few cases the mutants could not be regrown from permanent cultures, therefore, they were regenerated as described (Gäbel et al., 2013) using the oligonucleotides listed in Supplementary Table S1. All overproduction strains have been generated in this study (see below).

*Haloferax volcanii* strains were grown in complex medium with 50μg/ml uracil as previously described (Dambeck and Soppa, 2008). The cultures were grown in Erlenmeyer flasks at 42°C with shaking at 250rpm. Growth was either measured spectroscopically at 600nm or cells were counted using a Neubauer counting chamber.

The *E. coli* strain XL1-blue MRF’ (Agilent Technologies, Waldbronn, Germany) was used for cloning. It was grown in complex SOB medium under standard conditions (Hanahan, 1983).

### Generation of Overproduction Strains

For overproduction of proteins, the respective genes were cloned into the shuttle vector pSD1/R1-6 under the control of a strong synthetic constitutive promoter (Danner and Soppa, 1996). The genes were amplified using the primers listed in Supplementary Table S2 using genomic DNA from *H. volcanii* as template. The primers added the sequences for an N-terminal hexahistidine tag to the genes. The plasmids were isolated from *E. coli* and the sequences were verified before they were used to transform *H. volcanii*. All non-essential aIFs were overproduced in the respective deletion stains, while all essential aIFs were over-produced in the strain H26 Δ1279 that was used as wild-type strain concerning all proteins of this study. Table 1 gives an overview of the overproduced aIFs and the production strains. The dihydrofolate reductase (DHFR) was used as a negative control protein that is not involved in translation initiation. All subunits of the RNA polymerase were assumed to be essential without any testing, and, therefore, they were over-produced in the wildtype strain H26 Δ1279 (Table 2). All strains with expression plasmids derived from pSD1 were grown in the presence of Novobiocin (0.5μg/ml).

**Table 1:** Overview of aIF-genes and strains used in this study.

Name in this study	Gene ID	Production-Strain	Accession	MW [kDa]	Gene Name (Halolex)	Protein Name (Halolex)
dhfr	HVO_1279	WT	L9UT07	18.0	hdrA, folA1	dihydrofolate reductase
aIF1	HVO_1946	WT	D4GTH5	11.0	tif1a	translation initiation factor aIF-1 (SUI1 protein, bacterial-type IF3)
aIF1A1	HVO_0136	WT Δ0136	D4GZ79	11.5	tif1A1	translation initiation factor aIF-1A
aIF1A2	HVO_A0637	WT ΔA0637	D4GRU5	11.2	tif1A2	translation initiation factor aIF-1A
aIF2α	HVO_0699	WT Δ0699	D4GT46	29.5	tif2a	translation initiation factor aIF2 alpha subunit
aIF2β-1	HVO_1678	WT Δ1678	D4GZP2	15.0	tif2b1	translation initiation factor aIF2 beta subunit
aIF2β-2	HVO_2242	WT Δ2242	D4GVV8/L9VAS4	22.2	tif2b2	translation initiation factor aIF2 beta subunit / probable RNA-binding protein
aIF2γ	HVO_1901	WT	D4GTD4	44.0	tif2c	translation initiation factor aIF2 gamma subunit
aIF2Bsu	HVO_1934	WT Δ1934	D4GTG3	43.2	–	NUDIX family hydrolase/eIF-2B domain protein
aIF2Bα	HVO_0966	WT	L9USK7	35.0	–	ribose-1,5-bisphosphate isomerase
aIF2Bδ	HVO_2706	WT Δ2706	D4GW08/L9V7F9	30.8	–	eIF-2B domain protein
eIF4A-homolog	HVO_1333	WT Δ1333	D4GXK1/L9UST9	104.5	lhr2	ATP-dependent DNA helicase
aIF5A	HVO_2300	WT	D4GWG6/L9V7A1	14.2	tef5A	translation elongation factor aEF-5A
aIF5B	HVO_1963	WT	D4GTJ2	65.4	tif5B	translation initiation factor aIF-5B (bacterial-type IF2)
aIF6	HVO_0117	WT	D4GYW3/L9UI67	23.0	tif6	translation initiation factor aIF-6

*WT=H26 Δ HVO_1279.*

**Table 2:** Overview of rpo-genes and strains used in this study.

Name in this study	Gene ID	Production-Strain	Accession	MW [kDa]	Gene Name (Halolex)	Protein Name (Halolex)
rpoA1	HVO_0349	WT	D4GZX6	108.8	rpo1n, rpoA1	DNA-directed RNA polymerase subunit Rpo1N
rpoA2	HVO_0350	WT	D4GZX7	46.1	rpo1c, rpoA2	DNA-directed RNA polymerase subunit Rpo1C
rpoB1	HVO_0348	WT	L9UJM2	67.7	rpo2c, rpoB1	DNA-directed RNA polymerase subunit Rpo2C
rpoB2	HVO_0347	WT	L9UK99	58.9	rpo2n, rpoB2	DNA-directed RNA polymerase subunit Rpo2N
rpoD	HVO_2781	WT	L9V5W2	28.1	rpo3, rpoD	DNA-directed RNA polymerase subunit Rpo3
rpoH	HVO_0346	WT	D4GZX3	8.5	rpo5, rpoH	DNA-directed RNA polymerase subunit Rpo5
rpoL	HVO_1042	WT	D4GVL8	10.4	rpo11, rpoL	DNA-directed RNA polymerase subunit Rpo11

*WT=H26 Δ HVO_1279.*

### Characterization of Growth Curves

Growth curves were generated for the wild-type, for all deletion mutants, for all production strains with expression plasmids, and, as controls, for all respective strains containing the empty vector. In each case, exponentially growing pre-cultures were used to inoculate the test cultures. For each condition, 150μl medium was inoculated in triplicates in 96-well plates to an OD_600_ of 0.05. The outermost wells were filled with 1M NaCl to inhibit evaporation from the inner wells containing the test cultures. The OD_600_ was determined frequently using a microtiter plate photometer (Spectramax 340, Molecular Devices). Average values and their standard deviations were used to generate growth curves.

### Co-affinity Isolation of Proteins

*Haloferax volcanii* production cultures were grown overnight in complex medium, and the exponentially growing cells were harvested by centrifugation (4,700rpm, 30min., 4°C). The pellet was suspended in 4ml of ice-cold binding buffer (2.1M NaCl, 20mM HEPES, 20mM imidazole) and the cells were lysed by sonication on ice (3×30s, 50% duty cycle, output strength three). The lysate was subsequently centrifuged to remove cell debris and membranes (13,000rpm, 30min., 4°C) and to generate a cytoplasmic extract. 30μl aliquots were removed for analysis by SDS PAGE, the remaining supernatants were used for co-affinity isolation.

To this end, 500μl 50% Nickel Chelating Sepharose^®^ Fast Flow beads (NCS, GE Healthcare) were pelleted and resuspended in 1ml 0.2M NiCl_2_ solution. After incubation for 5min, the NCS was pelleted (13,000rpm, 30s.), washed three times in aqua bidest., and suspended in binding buffer (2.1M NaCl, 20mM HEPES, 20mM imidazole).

Two hundred and fifty micro liter of 50% NCS was pelleted and resuspended in 1.6ml cytoplasmic extract and incubated at room temperature with mixing to enable binding of protein complexes *via* his-tagged bait proteins. The NCS was pelleted, and an aliquot was removed from the supernatant for SDS-PAGE analysis of unbound proteins. The beads were washed four times with 1.6ml wash buffer (2.1M NaCl, 20mM HEPES, 30mM imidazole). Bound proteins were eluted by the incubation of the NCS in 0.1ml elution buffer (2.1M NaCl, 20mM HEPES, 700mM imidazole). After centrifugation and removal of the supernatant, a second elution step was performed with 0.1ml elution buffer. The eluates were dialyzed against 25mM Tris/HCl, pH7.2 on 13mm plates (Merck) or using a MEMBRA-CEL^®^ 3.5kDa tube. Aliquots representing all steps of the co-affinity isolation procedure were analyzed by SDS-PAGE. For normal-sized proteins standard SDS-PAGE was used, for small proteins below 10kDa Tricine-SDS-PAGE was used instead (Jiang et al., 2016). Suitable elution fractions were used to identify the protein composition by peptide mass fingerprinting and MS–MS analyses.

### Identification of Proteins by Mass Spectrometry

Samples were digested by the addition of Sequencing Grade Modified Trypsin (Serva) and incubated at 37°C overnight.

Peptides were desalted and concentrated using Chromabond C18WP spin columns (Macherey-Nagel, Part No. 730522). Finally, peptides were dissolved in 25μl of water with 5% acetonitrile and 0.1% formic acid.

The mass spectrometric analysis of the samples was performed using an Orbitrap Velos Pro mass spectrometer (ThermoScientific). An Ultimate nanoRSLC-HPLC system (Dionex), equipped with a custom end-fritted 50cm × 75μm ID C18 RP column filled with 2.4μm beads (Dr. Maisch) was connected online to the mass spectrometer through a Proxeon nanospray source. 1–15μl (depending on peptide concentration and sample complexity) of the tryptic digest were injected onto a 1cm×300μm ID C18 PepMap pre-concentration column (Thermo Scientific). Automated trapping and desalting of the sample was performed at a flowrate of 6μl/min using water/0.05% formic acid as solvent.

Separation of the tryptic peptides was achieved with the following gradient of water/0.05% formic acid (solvent A) and 80% acetonitrile/0.045% formic acid (solvent B) at a flow rate of 300nl/min: holding 4% B for 5min, followed by a linear gradient to 45% B within 30min and linear increase to 95% solvent B in additional 5min. The column was connected to a stainless steel nanoemitter (Proxeon, Denmark) and the eluent was sprayed directly towards the heated capillary of the mass spectrometer using a potential of 2,300V. A survey scan with a resolution of 60,000 within the Orbitrap mass analyzer was combined with at least three data-dependent MS/MS scans with dynamic exclusion for 30s either using CID with the linear ion-trap or using HCD combined with orbitrap detection at a resolution of 7,500. Data analysis was performed using Proteome Discoverer 2.2 (ThermoScientific) with SEQUEST search engine.

The identification of the protein compositions in elution fractions was performed for 14 aIFs and 7 subunits of the RNA polymerase. Two different negative controls were included, i.e., (1) *H. volcanii* cultures containing the empty vector, and (2) cultures overproducing the metabolic enzyme DHFR. The bioinformatic workflow for the removal of contaminants and false positives and identification of proteins that specifically bind to the overproduced aIFs or Rpos is discussed in the Results section.

### Generation of Phylogenetic Trees

After the genome of *H. volcanii* had been sequenced (Hartman et al., 2010), HVO_0966 was annotated to be a subunit of the translation initiation factor aIF2B. Later, the annotation was changed to the metabolic enzyme ribose-1,5-bisphosphate isomerase (R15BI). The three regulatory subunits of the eukaryotic translation initiation factor eIF2B (eIF2Bα, eIF2Bβ, eIF2Bδ) are homologous to one another and to a family of sugar phosphate isomerases methylthiophosphoribose isomerases (MTPI). It was decided to analyze the phylogeny of HVO_0966 with the aim to find indications whether it is more likely to be an aIF2B subunit than a metabolic enzyme.

BLAST searches at the website of the European Bioinformatics Institute were used to retrieve, in total, 34 sequences of proteins that are homologous to HVO_0966.^1^ At first, the taxonomic subset “human” was searched with HVO_0966, and the human sequences of eIF2Bα, eIF2Bβ, eIF2Bδ and of MTPI were retrieved. BLAST searches with these four protein sequences in the phylogenetic subsets “mammals,” “rodents,” “arthropoda,” “plants,” and “fungi” were used to retrieve the most similar non-human homologue of each group. Thereby, 24 eukaryotic sequences were retrieved (including the human proteins). In addition, the four human proteins were used for BLAST searches in the taxonomic subset “archaea,” to retrieve archaeal proteins that are similar to the four human protein families. At last, HVO_0966 was used for a BLAST search in the taxonomic subset of “archaea,” and proteins from different phylogenetic groups of archaea were retrieved, yielding a total set of 35 proteins (including HVO_0966).

The program “MEGA X” (Kumar et al., 2018) was used to generate a multiple sequence alignment (MSA) and calculate phylogenetic trees. All protein sequences were loaded individually into the program, and an MSA was generated. MEGA X allows to visualize and edit the MSA. This was used to remove positions that are phylogenetically un-informative, e.g., the N-terminal region that is exclusively present in the eIF2Bδ subfamily as well as insertions that are present in only one or very few sequences and that are obviously non-conserved. The resulting MSA was used to calculate three phylogenetic trees, which are based on the Maximum Likelihood, the Neighbor Joining, and the Maximum Parsimony algorithm. In each case 1,000 bootstrap replications were performed, and the fractions that were retrieved at each node were written to the respective nodes (% values). Capital letters were added to selected nodes to facilitate the discussion in the Results and Discussion sections.

### Databases and Programs

Bioinformatic analyses of the *H. volcanii* genome were performed at the website Halolex (Pfeiffer et al., 2008). The Halolex database is freely available, but currently usage is restricted to registered users. To request access, send a mail to halolex@rzg.mpg.de. The Integrated Genome Browser (Freese et al., 2016) was used to visualize the genome annotation, as well as the results of the dRNA-Seq study (Babski et al., 2016) and a recent RNA-Seq study (Laass et al., 2019). The program “Clone Manager”^2^ was used for the design of primer sequences and cloning experiments. The EMBL-EBI website^3^ was used for BLAST searches and to retrieve protein sequences.

## Results

### The Co-affinity Isolation Approach: Experimental Design and Data Analysis

The aim of this study was to unravel the protein–protein interaction network of translation initiation factors from the halophilic archaeon *H. volcanii*. In total, 14 genes are annotated in the genome of *H. volcanii* to encode (subunits of) translation initiation factors, which are summarized in Table 1. In a previous study, it was attempted to delete all these genes (Gäbel et al., 2013). Nine single-gene *in frame* deletion mutants could be successfully generated, while five genes turned out to be essential. In the present study, eight of the nine deletion mutants were used as background strains for the overproduction of the respective initiation factor. The remaining five proteins and one additional protein were overproduced in the wild-type. Supplementary Figure S1A gives an overview of the experimental workflow of co-affinity isolation. After overproduction of his-tagged versions of the proteins, cells were harvested and re-suspended in a high salt solution. Haloarchaea use the so-called salt-in strategy for osmotic adaptation, and the cytoplasmatic salt concentration equals that of the high-salt environment. Therefore, the co-affinity purification has to be performed under the native high salt conditions to prevent the dissociation of protein complexes and the unfolding of proteins, which is a typical problem when dealing with haloarchaeal proteins under low salt conditions.

**Figure 1:**
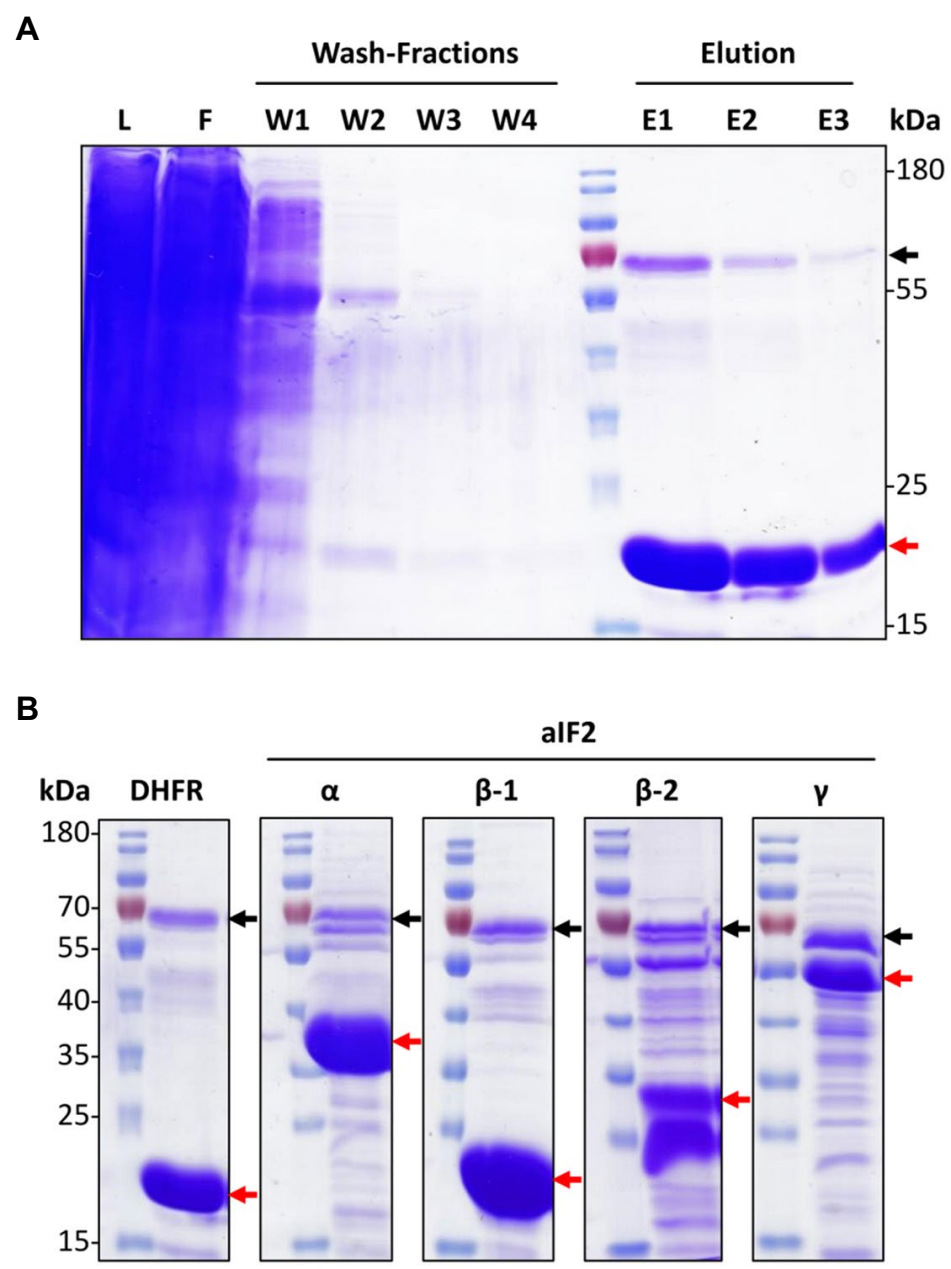
Overproduction and co-affinity purification of N-His_6_-tagged proteins. **(A)** SDS-Page representing different steps of the co-affinity purification of the negative control protein N-His_6_ DHFR. The red arrow points to N-His_6_ DHFR, the black arrow to an endogenous histidine-rich protein of *H. volcanii*, PitA. L – lysate, F – flow through, W – washing fractions, E – elution fractions. **(B)** SDS-Page of the elution fraction 1 from the co-affinity purifications of N-His_6_-tagged DHFR and the four indicated aIF2 subunits. Black and red arrows point to PitA and the produced His_6_-tagged respective aIF2 subunits. The figure shows one typical example of three biological replicates for each protein.

The cells were lysed by sonication, cell debris was removed by centrifugation, and nickel chelating sepharose was used for the affinity purification of his-tagged proteins. Figure 1A gives an overview of the affinity isolation of the DHFR. The major band in the elution fractions was the DHFR, showing that overproduction and affinity purification were successful (see red arrow in Figure 1A). A second protein of around 70kDa was also highly enriched (black arrow in Figure 1A). This is PitA, a native *H. volcanii* protein with a histidine-rich N-terminus, which binds with high affinity to the nickel chelating sepharose (Allers et al., 2010). Co-purification of PitA could be prevented by replacing the gene with a variant lacking the histidine-rich stretch (Allers et al., 2010). However, in this study we used PitA as an internal control for the success of the affinity isolation.

The same workflow was used for the affinity purification of the 14 aIFs. In all cases, three biological replicates were performed. A strain containing an empty vector was used as a second negative control in addition to the DHFR overproduction strain. Therefore, in total 48 cultures were used for the overproduction and affinity purification of the 14 aIFs and the two negative controls. In all cases, purification gels like the example shown in Figure 1A were used to guarantee that the last wash fraction was protein-free, and to estimate the pattern of co-purified proteins in the elution fractions. Typical elution fractions, representing one of the three biological replicates, are shown in Figure 1B for the four subunits of aIF2 and in Supplementary Figure S2 for all other aIFs.

The elution fractions containing the bait proteins and the mixtures of co-isolated proteins were dialyzed against a low salt buffer to enable downstream analyses. The protein mixtures after co-affinity isolation were identified by peptide mass fingerprinting. The proteins were digested with trypsin, and LC–MS/MS was used to analyze the resulting peptide mixtures. The peptide masses were used to search an *in silico* peptide library that was generated based on a genome annotation of *H. volcanii* that was supplied by Friedhelm Pfeiffer (MPI of Biochemistry, Martinsried, Germany). Result lists of identified proteins were obtained, which were sorted by the parameter “peptide spectrum matches” (PSM). The PSMs are semi-quantitative approximations of the amounts of co-isolated proteins, which are influenced by the affinities between bait and co-isolated proteins and the intracellular concentrations of the proteins.

Supplementary Figure S1B gives an overview of the bioinformatic workflow that was used for the identification of proteins that were specifically co-purified with the 14 aIFs of *H. volcanii*. In short, all proteins not encoded in the genome of *H. volcanii* were removed, e.g., trypsin and contaminations with human proteins. Then all proteins were removed that had not at least two “unique peptide hits.” Proteins that were identified in one or both of the two negative controls were also removed, including PitA and other proteins that can directly bind to the nickel chelating sepharose. Next, all proteins were removed that were not found in all three biological replicates. This workflow generated lists of trusted proteins that were specifically co-purified with aIFs. It should be noted that co-purification can be based on direct physical interactions, but can also be indirect based on bridging proteins or RNAs. An RNase step, which is sometimes included in the analyses of protein interaction networks, was deliberately not included in our co-affinity purification workflow. The project aimed at characterization of translation initiation networks, which require the presence of mRNAs as well as ribosomes. To facilitate further analyses, metabolic enzymes and hypothetical proteins were also removed, and, thereby, the analyses were concentrated on proteins involved in the biological processes translation, transcription, replication and repair, RNA and protein turnover, and protein folding. In the following paragraphs, various aspects of the results from co-affinity purification with the 14 haloarchaeal aIFs are discussed.

### The Ternary Initiation Factor aIF2

The heterotrimeric translation initiation factor aIF2 is of exceptional importance for translation initiation, because it brings the initiator tRNA to the P-site of the ribosome. All three subunits of the eukaryotic homolog eIF2 in yeast are essential. Unexpectedly, in *H. volcanii* only the γ-subunit is essential, while the deletion mutant missing the α-subunit is viable (Gäbel et al., 2013). *H. volcanii* contains two paralogous genes for the β-subunit, which can be individually deleted. However, a double mutant could not be obtained, and, thus, also the β-subunit is essential.

**Figure 2:**
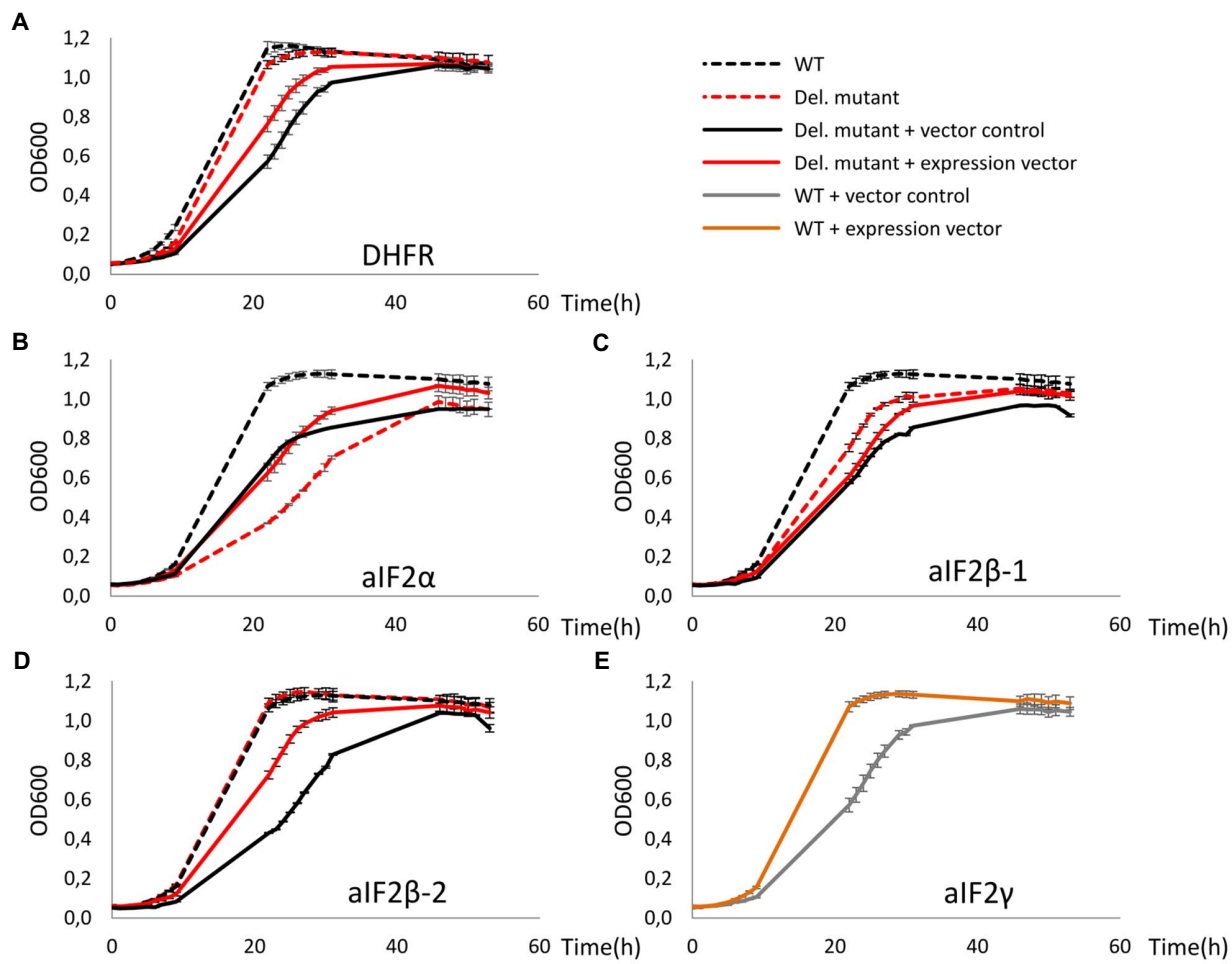
Growth curves of deletion mutants of *dhfr* and genes for aIF2 subunits as well as of the respective overproduction strains. All cultures were grown in triplicates under optimal growth conditions in complex medium in 96-well plates. The OD600 was measured frequently, and average values and standard deviations are shown. **(A-D)** Growth curves of the wildtype are shown in black, growth curves of the deletion mutants are shown in red. Dotted lines – vector-free cultures (medium without antibiotic), solid lines – vector-containing cultures (medium with 0.5μg/ml Novobiocin). The missing proteins in the respective deletion mutants are indicated at the bottom. **(E)** The essential aIF2 γ-subunit was overproduced in the wild type. Gray – wildtype with the empty vector, orange – wildtype with the expression plasmid.

The three deletion mutants as well as the *dhfr*-deletion mutant were transformed with expression plasmids containing the respective genes, and the wild-type was transformed with an expression plasmid for the overproduction of the γ-subunit. As a control, all strains were also transformed with the empty vector. Figure 2 shows growth curves of all plasmid-lacking and plasmid-containing strains. The plasmid-free cultures (dotted lines) were used to verify that the deletion mutants had the same phenotypes as previously reported (Gäbel et al., 2013). And indeed, deletion of *aIF2α* resulted in a very severe growth defect (Figure 2B), in contrast to the deletion of the genes for either of the two beta subunits (Figures 2C,D), and also deletion of *dhfr* did not compromise growth (Figure 2A). In all cases, the presence of the empty vector in the wildtype (black solid lines) led to a growth defect in comparison to the vector-free cultures (black dotted lines). Obviously, the Novobiocin resistance gene on the vector could not fully restore growth in Novobiocin-containing medium to the level in antibiotic-free medium. Therefore, growth characteristics of plasmid-free and plasmid-containing strains cannot be compared, and production strains have to be compared with the respective controls containing the empty vector. Overproduction of aIF2α and of aIF2β-1 did not increase (or decrease) the growth rate, but resulted in a slight increase in growth yield (Figures 2B,C). In contrast, overproduction of aIF2β-2 and of aIF2γ increased the growth yield considerably (Figures 2C,E). Because aIF2γ was overproduced in the wildtype, this indicates that the native concentration of aIF2γ might be rate-limiting.

Next, all four tagged-subunits were used for co-affinity purification, as described above. The patterns of co-isolated proteins are shown in Figure 1B in comparison to each other and the negative control producing DHFR. The following results were obtained: (1) a large number of proteins could be co-isolated with the aIF2 subunits, in contrast to the negative control, (2) the patterns of co-isolated proteins is different for the four aIF subunits, and (3) the number of co-isolated proteins is much higher for aIF2β-2 than for aIF2β-1. A higher importance of aIF2β-2 compared to aIF2β-1 is consistent with the growth analyses (Figures 2C,D) and with previous observations (Gäbel et al., 2013).

**Figure 3:**
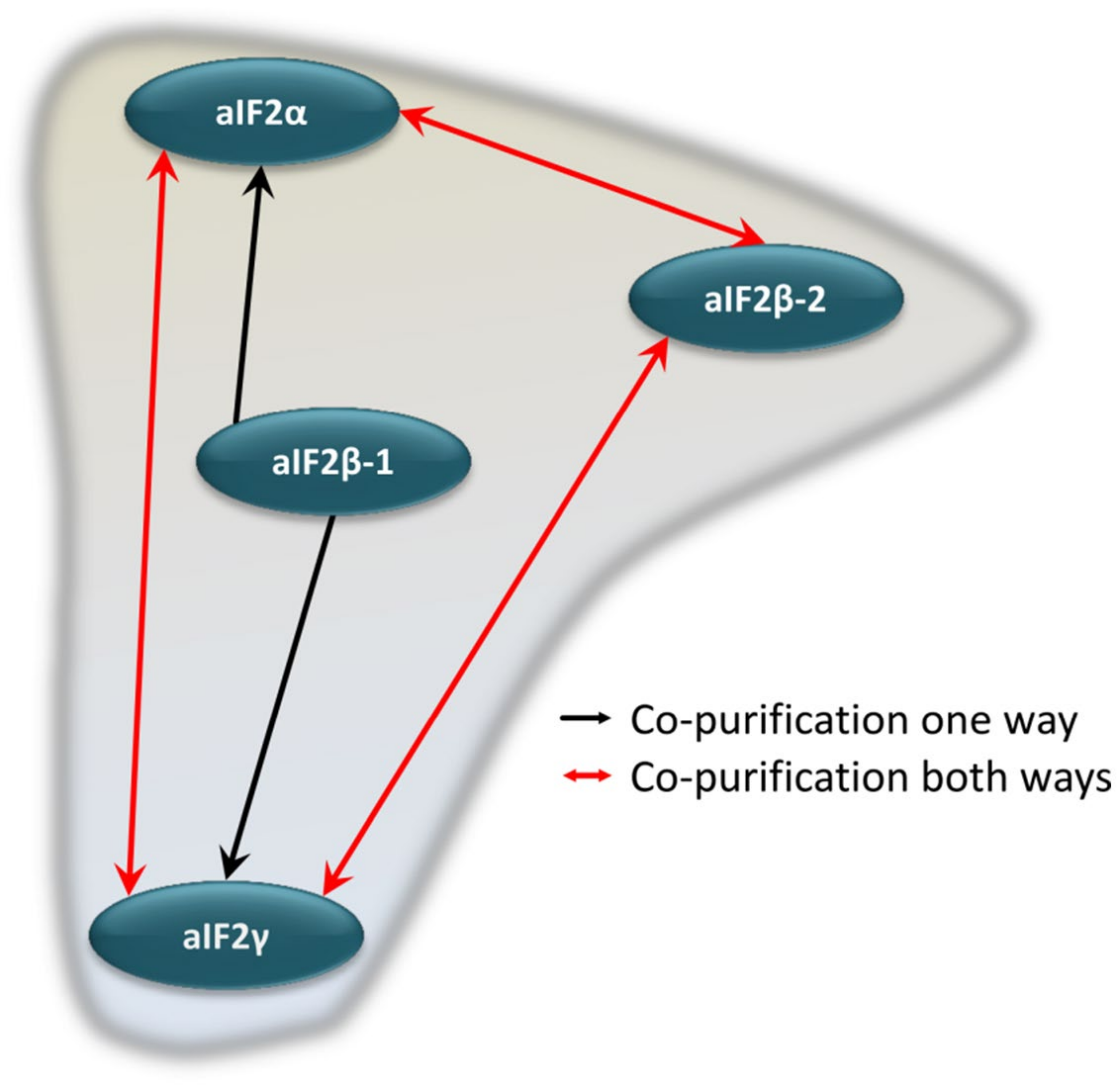
Schematic overview of the internal aIF2 subunit interaction network. Red arrows denote reciprocal co-purification, while black arrows depict one-directional co-purification.

The co-isolated proteins were identified by peptide mass fingerprinting, as described above. Importantly, reciprocal co-isolation between the three subunits aIF2α, aIF2β-2, aIF2γ was observed, indicating that the three proteins form a heterotrimeric complex, as in eukaryotes and other archaea. The subunit aIF2β-1 was not co-isolated with any of the other subunits. However, with aIF2β-1 as bait, aIF2α and aIF2γ were co-isolated, indicating that two alternative heterotrimeric aIF2 complexes exist in *H. volcanii* (Figure 3).

Further proteins that could be co-isolated with one or more of the aIF2 subunits are listed in Supplementary Table S3. Translation initiation occurs at the ribosome, therefore, it was not surprising that 14 ribosomal proteins were co-isolated. However, aIF1 and aIF1A, which are part of the archaeal preinitiation complex together with aIF2 (Coureux et al., 2020), were not co-isolated. A translation initiation factor that could be co-isolated was the universally conserved aIF5B (eIF5B in eukaryotes, and IF2 in bacteria). Another protein that could be co-isolated with aIF2α as well as with aIF2γ is HVO_0966, which is annotated as the enzyme R15BI. However, for reasons that are discussed below, we propose that in fact HVO_0966 is a translation initiation factor and should be renamed to aIF2Bα. In eukaryotes, eIF2B is a regulator and GDP/GTP exchange factor for eIF2.

Unexpectedly, also several proteins could be co-isolated with aIF2 that are involved in transcription initiation, transcription regulation, and DNA repair. The relevance of these results will be discussed in the Discussion section.

### The aIF–aIF Interaction Network

**Figure 4:**
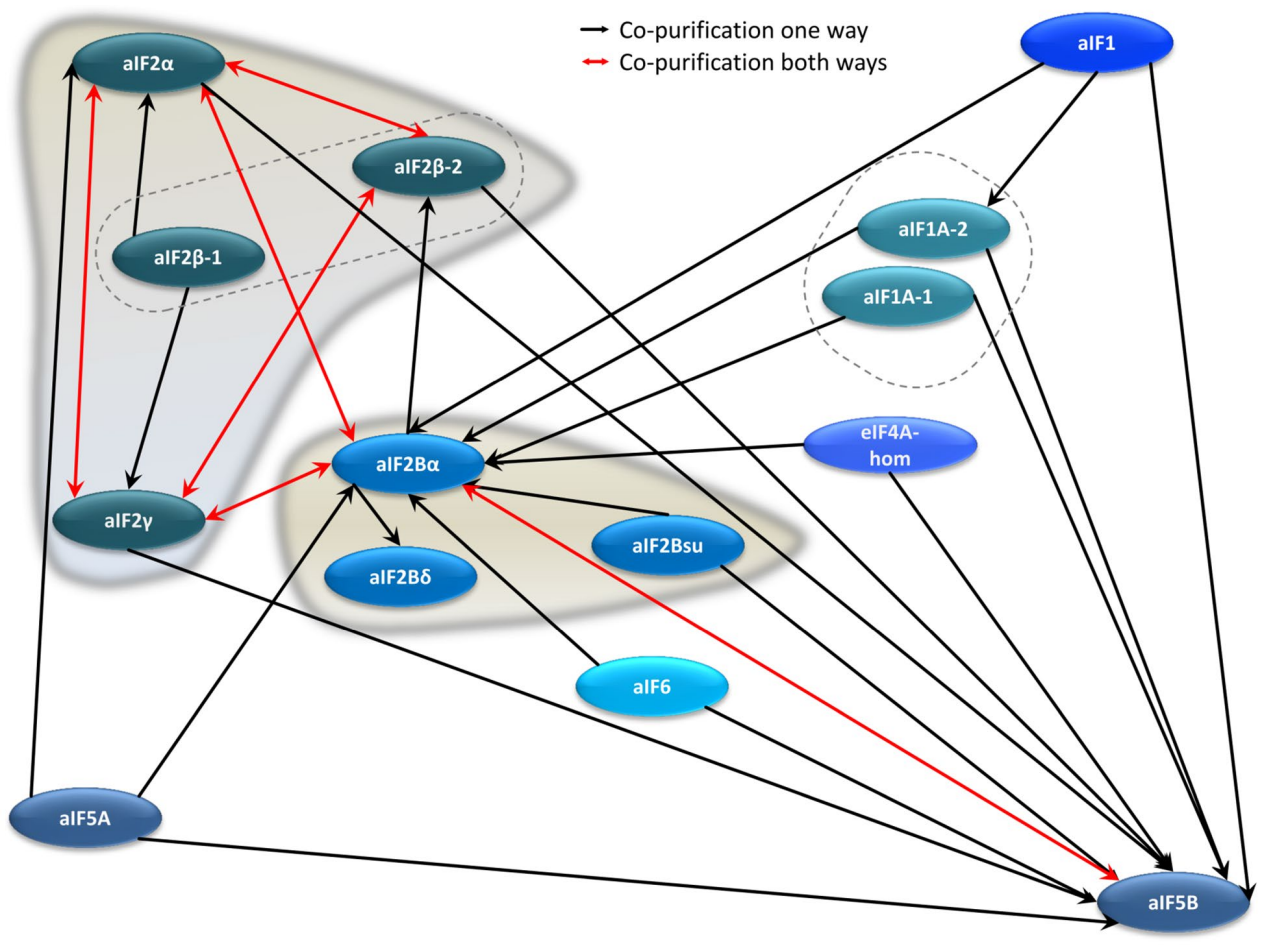
Schematic overview of the aIF–aIF interaction network. Red arrows denote reciprocal co-purification, while black arrows depict one-directional co-purification.

Co-affinity isolations to identify interaction partners were performed with ten further translation initiation factors, in addition to the four aIF2 subunits. This allowed to unravel the aIF–aIF protein interaction network. The results are summarized in Figure 4 and in Supplementary Table S4. The growth curves of all deletion mutants and production strains are shown in Supplementary Figure S3. Most interactions were observed only in one direction, however, in six cases co-isolation occurred in both directions, further underscoring their importance (red double arrows in Figure 4). Two hubs with many interactions are clearly visible in the aIF–aIF interaction network, i.e., aIF5B and aIF2Bα (HVO_0966). The universally conserved factor aIF5B could be co-isolated by 11 other aIFs, while aIF5B as bait led only to the co-isolation of aIF2Bα. At first sight this asymmetry seems to be unexpected, and reciprocal co-isolation should be expected whenever two proteins interact. However, notably, the co-isolation experiments with two proteins are not symmetrical, i.e., only the bait protein is overproduced, while the co-isolated interaction partner has its native intracellular concentration. In addition, only the bait protein carries an N-terminal tag, which might interfere with complex formation. For example, if the N-terminus of aIF5B would be important for the interaction with many other proteins, the N-terminal tag would inhibit co-isolation with aIF5B as bait, while the untagged aIF5B can easily be co-isolated with tagged other proteins as baits.

The second major hub in the aIF–aIF interaction network is the protein that we would like to re-annotated to aIF2Bα (HVO_0966), albeit it is currently annotated as an enzyme. It was co-isolated with 10 different aIFs, while with aIF2Bα as bait co-isolated five aIFs, including two subunits of aIF2. This very central position of HVO_0966 in the aIF–aIF interaction network prompted us to study its phylogeny, with the aim to unravel its connections to other archaeal and eukaryotic initiation factors and enzymes (see below).

Two genes in the *H. volcanii* genome are annotated to encode putative subunits of aIF2B, i.e., aIF2Bδ (HVO_2706) and aIF2Bsu (HVO_1934). In eukaryotes, eIF2B is composed of two subcomplexes, the subunits eIF2Bα, eIF2Bβ, and eIF2Bδ form a regulatory subcomplex, while the subunits eIF2Bγ and eIF2Bε form a catalytic subcomplex (Bogorad et al., 2014). Genes for the catalytic subunits are not present in archaeal genomes, while often genes with similarities to the three regulatory subunits are present, so that the presence of a ternary complex in archaea has been postulated (Dev et al., 2009). However, our analysis of the aIF–aIF interaction network did not give strong support for the presence of such a ternary complex in *H. volcanii*. Reciprocal co-isolation was not observed between any of the three putative subunits, in contrast to the aIF2 ternary complex, and co-isolation between the putative aIF2Bδ and aIF2Bsu was not observed at all. In addition, in stark contrast to the many interactions of aIF2Bα, only one or two, respectively, interactions were found for the other two proteins. Taken together, these results argue against the existence of a heteromeric complex of the three proteins, at least in haloarchaea.

As discussed above, direct interactions between aIF1 and aIF1A and aIF2 were not found, however, aIF1 as well as both paralogs of aIF1A interact with the central hub protein aIF2Bα, which, in turn, interacts with aIF2. Therefore, an indirect interaction does exist within the aIF–aIF interaction network. In addition, a Cryo-EM structure of the preinitiation complex of *P. abyssi* revealed that the prominent interactions of aIF1, aIF1A, and aIF2 are formed with ribosomal proteins, and not with each other (Coureux et al., 2020).

### The Interaction Network Between aIFs and the Ribosome

**Figure 5:**
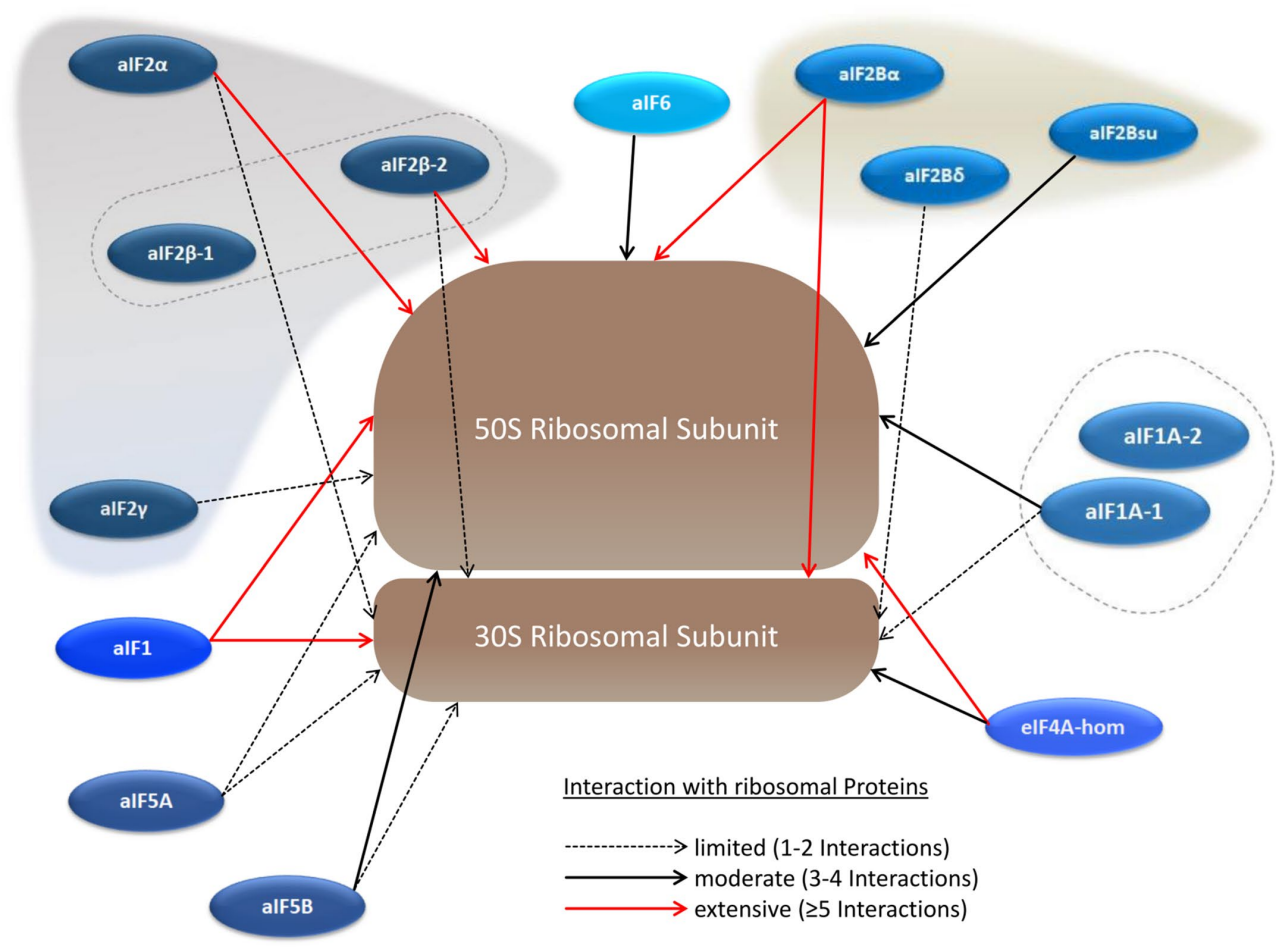
Schematic overview of the aIF–ribosome interaction network. The large and the small ribosomal subunits are shown schematically instead of including all ribosomal proteins individually. The numbers of co-purified ribosomal proteins are indicated by red arrows (at least 5 co-purified proteins), solid black arrows (3–4 co-purified proteins), and dotted black arrows (1–2 co-purified proteins).

Translation initiation occurs at the ribosome, and, therefore, many interactions between translation initiation factors and ribosomal proteins can be expected. And indeed, a large number of ribosomal proteins could be co-isolated with the 14 (subunits of) translation initiation factors as bait proteins. The results are summarized in Figure 5 and in Supplementary Table S5. The interaction with an aIF and the ribosome was defined as “extensive,” when at least five ribosomal proteins were co-isolated (red arrows in Figure 5). Based on this definition, two proteins had extensive interactions with the large as well as with the small ribosomal subunit. One of the proteins is aIF2Bα, which underscores that aIF2Bα is a central hub in the translation initiation protein interaction network. In contrast, the results are very different for the two proteins that are annotated as putative aIF2B subunits, again not strengthening the idea that a ternary aIF2B complex might exist in haloarchaea.

The other protein with extensive interactions with both ribosomal subunits is aIF1. In contrast, one of the two aIF1A paralogs has only limited to medium interactions, while not a single ribosomal protein was co-isolated with the other paralog, aIF1A-2, as bait. In the structure of the preinitiation complex of *P. abyssi* the interactions of both aIF1 and aIF1A with the ribosome seemed to be similar (Coureux et al., 2016).

As mentioned above, many ribosomal proteins were co-isolated with two subunits of aIF2 as baits. The interactions of both proteins were much more extensive to the large subunit, which is unexpected, because aIF2 is thought to leave the preinitiation complex before the large subunits joins and the elongation phase begins. The universally conserved factor aIF5B, which was found to be a central hub in the aIF–aIF network, led only to a limited to medium co-isolation of ribosomal proteins. Possible implications of the observed aIF–ribosome interactions are considered in the Discussion section.

### HVO_0966, an Important Translation Initiation Factor or a Metabolic Enzyme?

The results of the co-affinity isolation approach described above indicated that HVO_0966 is a central hub in the translation initiation protein interaction network. On the other hand, HVO_0966 is annotated as the enzyme R15BI. A BLAST search with HVO_0699 in the domain Archaea retrieved exclusively proteins that are also annotated as R15BI. In contrast, a BLAST search in the domain of Eukaryotes retrieved methylthioribose-1-phosphate isomerase as well as the alpha subunit of translation initiation factor eIF2B. A BLAST search in the domain of Bacteria retrieved methylthioribose-1-phosphate isomerase, with very few exceptions.

The annotation of the archaeal proteins is based on a publication by Sato et al., who proposed that in *T. kodakarensis* three enzymes convert AMP to two molecules of 3-phosphoglycerate, which is part of the central metabolism (Sato et al., 2007). This gave a biological function to the third enzyme, ribulose-1.5-bisphosphate carboxylase/oxygenase (RuBisCO; TK2290), the role of which in archaea had been enigmatic before. The second enzyme is R15BI (TK0185), which isomerizes ribose to ribulose, and thus yields the substrate for RuBisCO. Before, the protein had been annotated to be an aIF2B subunit, and later this and all homologous archaeal proteins were re-annotated to be R15BIs. In a later publication, the enzymatic function of TK0185 was verified and the enzyme kinetic characteristics were characterized (Aono et al., 2012).

*Haloferax volcanii* contains all three genes, and, thus, could also use this pathway to funnel AMP into the central metabolism. In addition, the genes for the first two enzymes are adjacent (HVO_0965 and HVO_0966), and the third gene is close by (HVO_0970). Analysis of results from a RNA-Seq and a dRNA-Seq study (Babski et al., 2016; Laass et al., 2019) revealed that all three genes have independent promoters, and that the transcript level of HVO_0970 is much higher than that of HVO_0966 and HVO_0965. Nevertheless, the existence and close neighborhood of the three genes indicates that HVO_0966 might have the enzymatic function of a R15BI, like the homolog from *T. kodakarensis.* Therefore, the MS results were checked whether the two other enzymes of the AMP salvage pathway were co-isolated with HVO_0966, but this was not the case. However, it should be noted that a successful co-isolation of the two other enzymes with HVO_0966 would have been a strong indication for the existence of the AMP salvage pathway also in *H. volcanii*, but that the lack of co-isolation does not indicate its absence, because enzymes of metabolic pathways often do not form heteromeric complexes, but work as independent modules.

Taken together, strong arguments for both putative functions of HVO_0966 exist. Therefore, we decided to generate phylogenetic trees to analyze the evolution of HVO_0966 and its archaeal and eukaryotic homologs, with the aim to clarify whether HVO_0966 is more likely to be a haloarchaeal translation initiation factor or an enzyme. The three regulatory subunits of the eukaryotic eIF2B are paralogs, and they are homologs to the enzyme MTPI. Therefore, the sequences of eIF2Bα, eIF2Bβ, eIF2Bδ and MTPI from humans and one representative of rodents, Arthropoda, plants and fungi were retrieved from protein sequence databases. In addition, BLAST searches were performed with HVO_0966 as well as with the four human proteins in the domain of Archaea, and the most similar archaeal sequences were retrieved irrespective of their annotation. In total, 35 sequences were used to generate a MSA. The MSA was manually edited to remove regions without phylogenetic information, e.g., non-conserved long N-termini of the β and δ subunits of eIF2B and insertions in single sequences. The resulting MSA was used to generate phylogenetic trees with MEGA X (Kumar et al., 2018). Three different approaches were used, i.e., Maximum Likelihood (ML), Neighbor Joining (NJ), and Maximal Parsimony (MP). In all three cases 1,000 bootstrap replications were performed to estimate the confidence of different parts of the tree.

**Figure 6:**
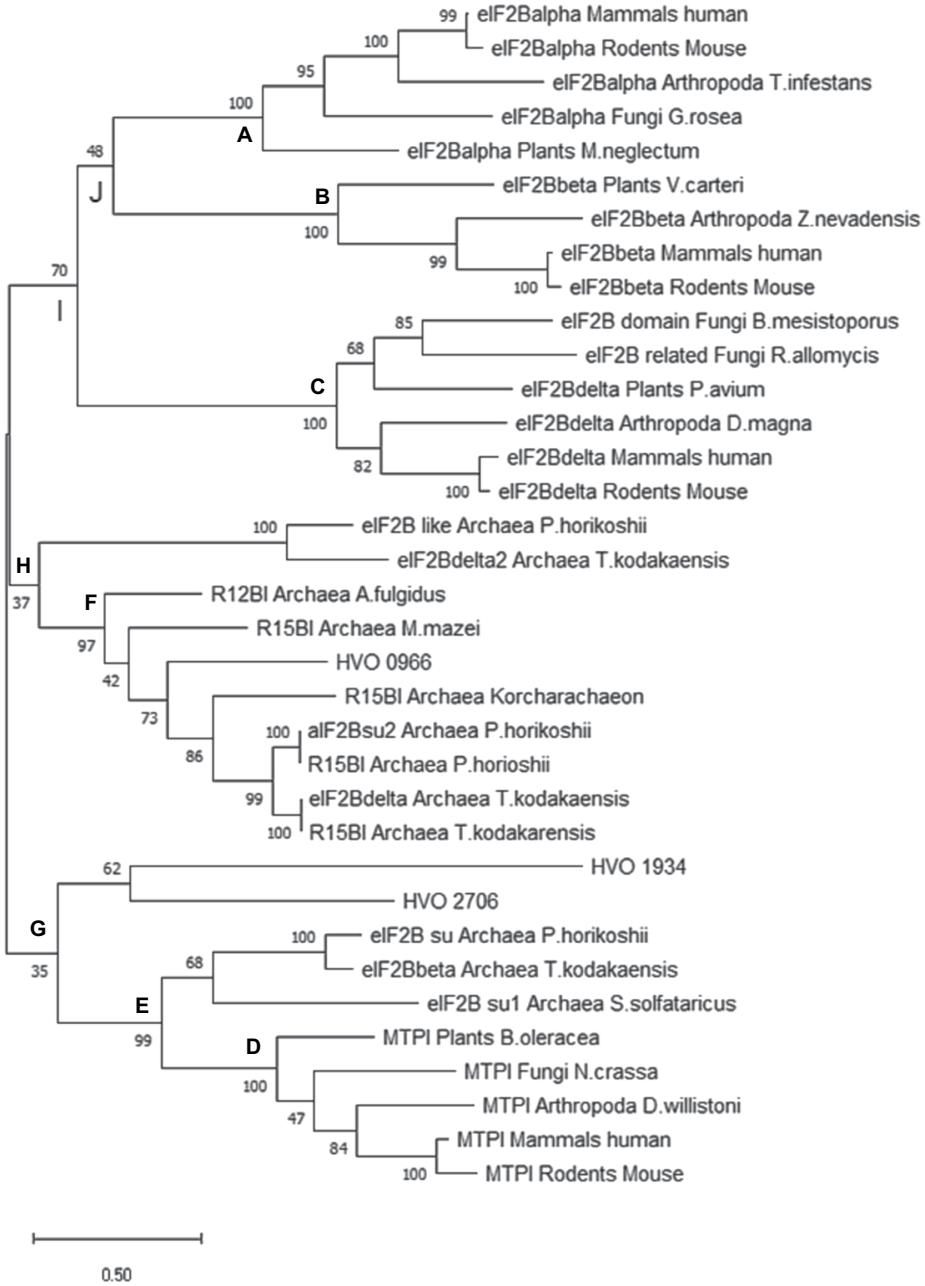
Phylogenetic tree of selected archaeal and eukaryotic proteins of the a/eIF2B superfamily. Criteria for selection of the 35 proteins are explained in the text. A maximum likelihood (ML) tree was calculated using the program Mega X (Kumar et al., 2018). 1,000 bootstrap replications were performed, and the bootstrap values are shown at each node (%). Specific nodes of interest were labeled with A to G to facilitate the discussion (see text).

The ML tree is shown in Figure 6, the NJ tree is shown in Supplementary Figure S4, and the MP tree is shown in Supplementary Figure S5. While the three trees are not identical, the following major results are the same for all three approaches: (1) the three eukaryotic regulatory eIF2B subunits form monophyletic groups with very high bootstrap support (nodes A-C in Figure 6). The only exception is one protein from fungi, which was retrieved with a BLAST search with the human eIF2Bβ, but is found in the δ subtree. (2) Also the five MTPIs form one monophyletic group with a very high bootstrap value (node D). (3) Three archaeal proteins group together with the eukaryotic MTPIs (node E). All three are annotated as homologs to eIF2B subunits, but might well be enzymes based on their phylogenetic relationship to MTPIs. (4) Eight archaeal sequences form one phylogenetic group with high bootstrap support (node F). It is tempting to speculate that all members of this group have the same function, in spite of the very mixed annotations. Clearly, the annotations of the archaeal proteins are as yet unresolved and do not help to understand their function. This phylogenetic group includes HVO_0966, which seems to be a central hub of the translation initiation network based on the results presented above, as well as protein TK0185 from *T. kodakarensis*, which was shown to be a R12BI. (5) The archaeal group is between the eukaryotic eIF2B groups and the eukaryotic MTPI group. All deep nodes (G, H, I, J) have very low bootstrap supports, therefore, the phylogenetic analysis does not help to decide whether HVO_0966 and the other members of the archaeal group are more likely to be enzymes or translation initiation factors. In the Discussion, we will argue that it is possible that members of this protein family could have both functions and are moonlighting proteins. (6) The other two *H. volcanii* proteins that are annotated to be putative eIF2B subunit homologs (HVO_1934, HVO_2706) cluster together and far from HVO_0966. Their position in the tree depends on the algorithm used and cannot be clarified.

### The Interaction Network of aIFs With Translation and Transcription Proteins

A full list of co-isolated proteins (after filtering, see Materials and Methods) is shown in Supplementary Table S6. In addition to the aIFs and the ribosomal proteins discussed above, many more proteins could be co-isolated. These included eight subunits of the RNA-polymerase (Rpo), two TATA box-binding proteins, and 27 transcription factors, indicating that translation initiation and transcription are not independent in *H. volcanii*. In bacteria, coupling of transcription and translation has been discussed for a long time, and, very recently, a direct interaction between RNA-polymerase and the ribosome has been reported (see Discussion). One experimental study exists, which indicated that coupling of transcription and translation might also occur in archaea (French et al., 2007). Electron microscopy was used to show that polysomes are close to DNA in lysed cells of *T. kodakarensis*. In this Special Issue of Frontiers in Microbiology, Weixlbaumer et al. propose that transcription and translation in archaea might be coupled, based on bioinformatic comparisons of bacterial and archaeal proteins (Weixlbaumer et al., 2021). To gain further insight into a putative coupling of transcription and translation in haloarchaea, we decided to study the interaction between the RNA polymerase and translation initiation factors further.

### The Interaction Network of RNA Polymerase Subunits

**Figure 7:**
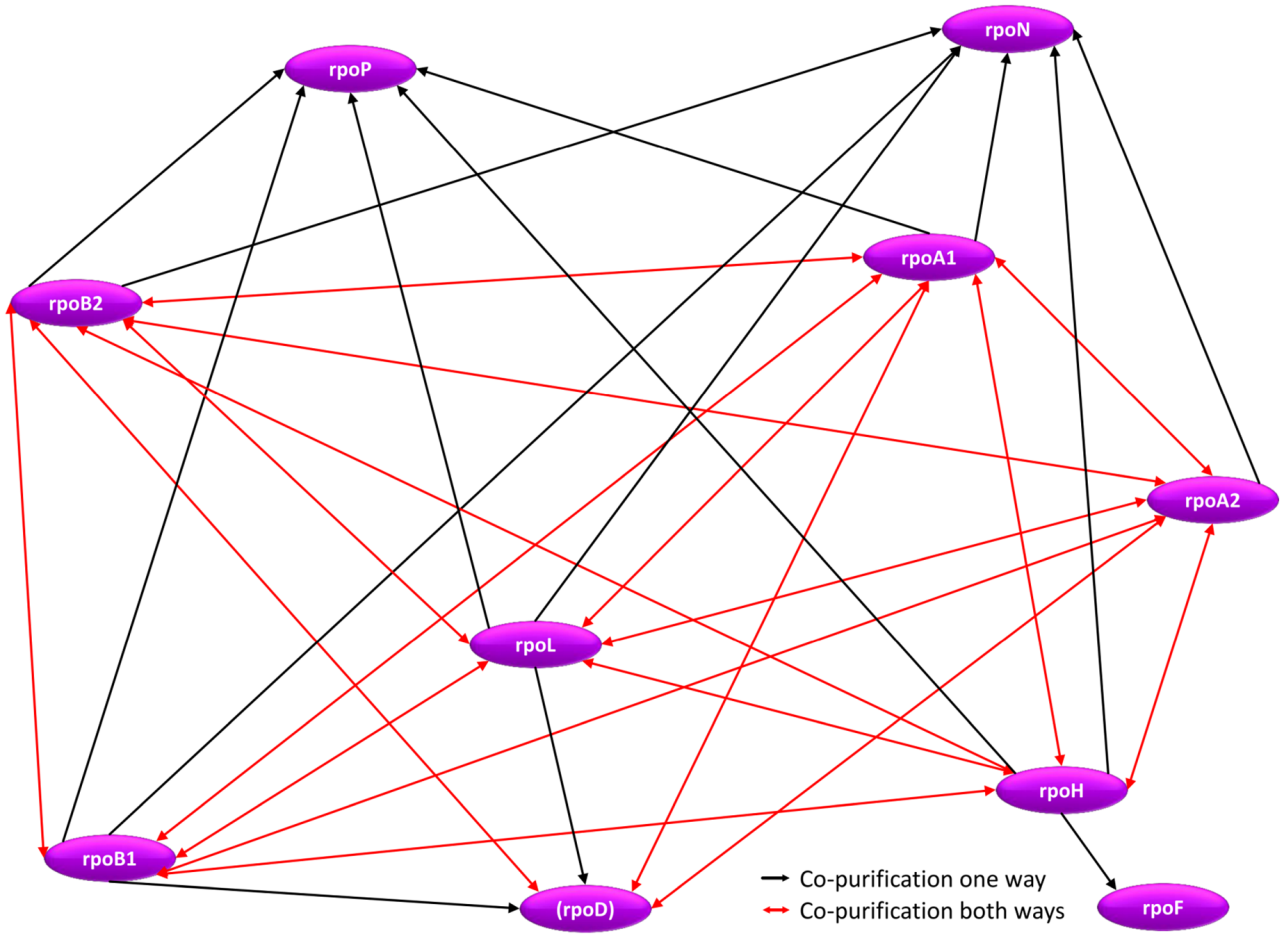
Schematic overview of the internal RNA polymerase subunit interaction network. Red arrows denote reciprocal co-purification, while black arrows depict one-directional co-purification.

The genes for seven subunits of the RNA polymerase were cloned into an expression vector, and the proteins were produced with an N-terminal hexahistidine tag (Table 2). All subunits of the catalytic core were included (RpoA1, RpoA2, RpoB1, RpoB2), two subunits of the assembly platform (RpoD, RpoL), and one auxiliary subunit (RpoH). Subunits of the RNA polymerase can be assumed to be essential, therefore, it was not attempted to generate deletion mutants, but the tagged proteins were produced in the wildtype, in addition to the native proteins encoded on the chromosome. In all seven cases co-isolation of other proteins turned out to be possible, showing that the tagged proteins were incorporated into the multi-subunit RNA polymerase complex. The interaction network between RNA polymerase subunits is shown in Figure 7 and in Supplementary Table S7. In nearly all cases, reciprocal co-isolation of the seven bait subunits was successful. Only the affinity isolation of RpoD did not result in the co-isolation of RpoB1 and RpoL, which can readily be explained by the very low production level of RpoD, in contrast to the other six subunits (see Supplementary Figure S6A). The effect of overexpression on the growth of *H.* volcanii is shown in Supplementary Figure S6B. Three further RNA polymerase subunits could be co-isolated with one or several of the bait subunits, i.e., the two assembly platform subunits RpoN and RpoP were co-isolated with six and five subunits, respectively, and the auxiliary subunit RpoF was co-isolated with RpoH. The only two subunits that were not co-isolated with any of the seven bait subunits were the two auxiliary subunits RpoE and RpoK. Possible reasons include loss of these two auxiliary subunits during the affinity purification procedure, which includes intensive washing before elution, and/or failure to detect the co-isolated subunits *via* peptide mass fingerprinting (RpoK is very small with only 58 amino acids). In any case, the results showed that the seven tagged bait proteins were integrated into the complex and enabled the affinity isolation of a (nearly) complete RNA polymerase, which should allow to unravel the RNA polymerase interaction network.

### The Interaction Network Between RNA Polymerase Subunits and aIFs

**Figure 8:**
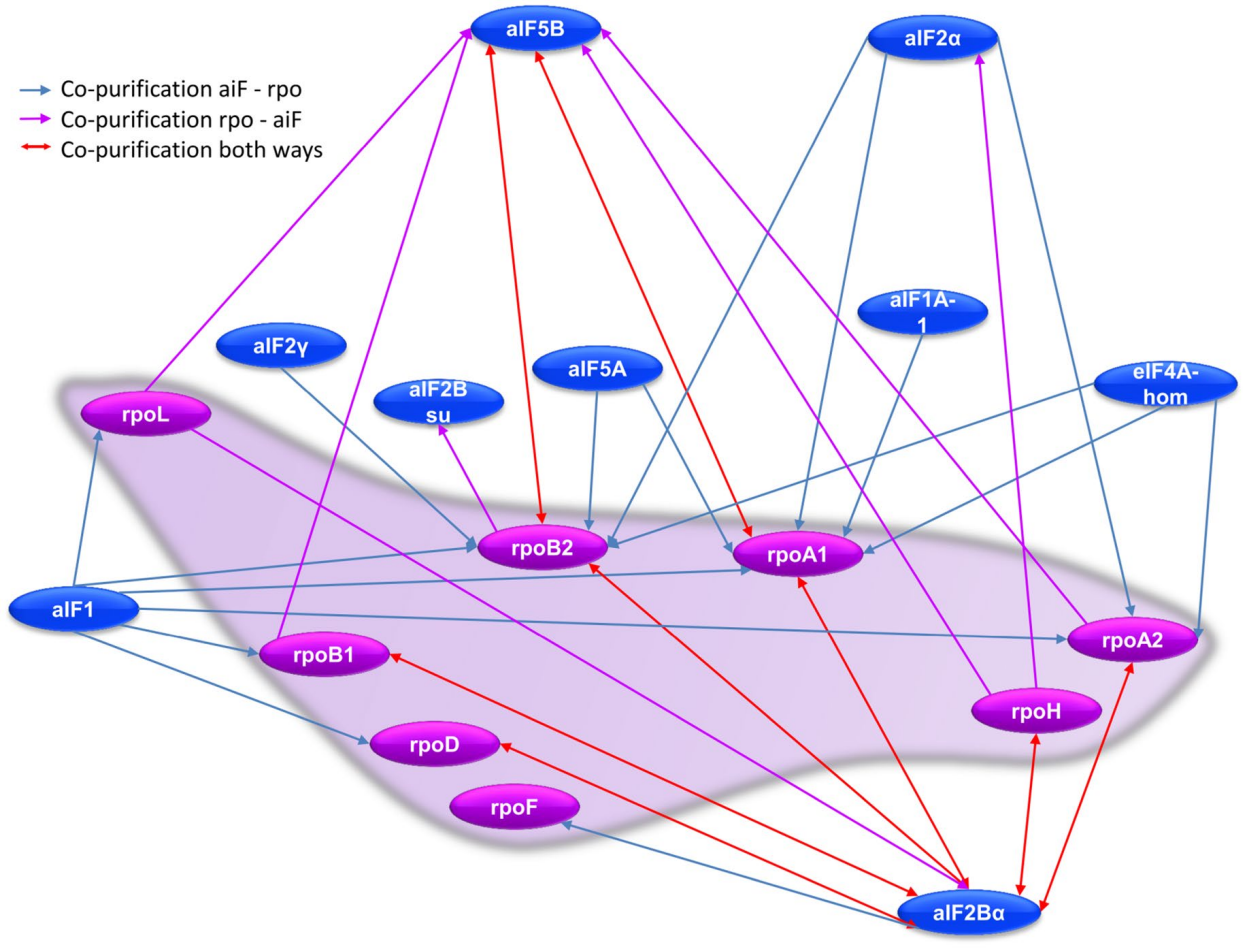
Schematic overview of the RNA polymerase subunit–aIF interaction network. Red arrows denote reciprocal co-purification, blue arrows indicate co-purification of a Rpo subunit with a tagged aIF bait, while purple arrows indicated co-purification of an aIF with a tagged Rpo subunit as bait.

Co-affinity isolations with 14 aIFs as bait proteins and seven Rpos as bait proteins enabled to gain a comprehensive view of the interconnections between RNA polymerase and translation initiation. The results are shown in Figure 8 and Supplementary Tables S8 and S9. Obviously, two interaction hubs exist, which are characterized by a high number of interactions and reciprocal interactions, i.e., aIF2Bα has eight interactions with Rpos, six of which are reciprocal, and aIF5B has six interactions, two of which are reciprocal. No other aIF shows a reciprocal interaction with a Rpo subunit. Only two additional aIFs are co-isolated with a Rpo as bait, namely aIF2α with RpoH and aIF2Bsu with RopB2. As discussed above, eight different aIFs as baits led to the co-isolation of one or more RNA polymerase subunits (Supplementary Table S9). Two possible reasons for an only uni-directional co-isolation have been discussed above, for an essential multi-subunit complex like the RNA polymerase two additional reasons apply: (1) The overproduction of one subunit does not lead to the overproduction of the whole complex, because all other subunits are encoded on the chromosome and produced under the control of the native promoters, and (2) only a subpopulation of complexes carries the tag and can be co-purified, which results in a lower concentration during the isolation and washing steps. In any case, the high number of (reciprocal) co-purifications between RNA polymerase subunits and the initiation factors aIF5B and aIF2Bα underscores that transcription and translation initiation are not independent processes in haloarchaea.

### Interactions of RNA Polymerase Subunits With Further Translation and Transcription Proteins

Many additional proteins could be co-isolated with RNA polymerase subunits as bait proteins, in addition to the four aIFs (Supplementary Table S10). Notably, this includes 21 ribosomal proteins, which – again – underscores the interdependence of transcription and translation in *H. volcanii*.

RNA polymerase subunits as baits also led to the co-isolation of one TATA box binding protein and of 21 transcription factors (TFs). Of these 21 TFs, 19 were also co-isolated with aIFs as baits, underlining that these are real positives and that they are present in the complexes that also contain aIFs and Rpos (Supplementary Table S10).

## Discussion

### Elucidation of Protein–Protein Interaction Networks *via* Co-affinity Isolation of Complexes

Most if not all biological processes rely on the interaction of biomolecules, e.g., proteins and nucleic acids. Therefore, experimental approaches to unravel single interactions or complete interaction networks are of utmost importance. Different aspects of experimental approaches to characterize protein–protein interaction networks have been reviewed recently (Koh et al., 2012; Yang et al., 2015).

Co-affinity isolation of protein complexes using bait proteins followed by the identification of co-isolated binding partners has been applied very extensively using model species of bacteria and eukaryotes. However, also a few projects have been reported that unraveled protein–protein interaction networks in archaea. For example, the DNA replication protein network of *T. kodakarensis* was analyzed using 19 hexahistidine-tagged replication proteins as baits (Li et al., 2010). In another study, the protein interaction network of the taxis signal transduction pathway of *Halobacterium salinarum* was studied using 18 tagged bait proteins (Schlesner et al., 2012). A third study analyzed the protein–protein network of genomic maintenance in *P. abyssi* with 22 tagged bait proteins (Pluchon et al., 2013). Here, we report the fourth study that used co-affinity isolation of protein complexes with tagged bait proteins coupled to MS analysis to unravel protein interaction networks in archaea.

Initially, three different tags were compared, which were fused to the DHFR, i.e., the hexahistidine tag (His_6_), the CBD tag, and the streptavidin tag. The His_6_ tag turned out to be highly superior to the other two tags, and consequently, it was used for all proteins analyzed in this study (Schramm and Soppa, unpublished results). In total, 14 aIFs, seven Rpo subunits, and the metabolic enzyme DHFR as negative control were tagged, affinity isolated, and co-isolated binding partners were identified. Three biological replicates were performed to guarantee reproducibility and minimize false-positive hits. Taken together, we present here one of only extremely few studies that unraveled a protein–protein interaction network in an archaeon, and with 22 analyzed proteins its size is second to none of the three previous studies, which concentrated on other biological processes.

The bait proteins were overproduced, as in the three other studies with archaea mentioned above and many studies with bacteria and eukaryotes. Notably, this will lead to the formation of non-native complexes with additional proteins, which have lower affinities to the bait proteins than the native interaction partners (false positives). However, cell disruption leads to a very large dilution of the cytoplasm, so that low affinity complexes will dissociate again, and only native high-affinity complexes will remain. In addition, we applied intensive washing to guarantee that all proteins that bind non-specifically to the bait proteins or to the nickel-chelating column were removed. If we had expressed the bait proteins at their native levels, the high dilution and stringent washing could have led to the dissociation of native protein complexes of intermediate affinity (false negatives). Taken together, we think that the experimental design of bait overproduction and omission of a crosslinking step has a high probability to keep the false-positive as well as the false-negative rate low.

It should be noted that the observed interactions are not restricted to direct physical binding partners, but also includes indirect interactions. For example, if a heteromeric complex exists, it can be expected that many or all subunits can be co-isolated, even those that do not directly physically interact with the bait protein. In the present case, translation initiation involves the mRNA as an essential constituent, and, therefore, also indirect protein–mRNA–protein interactions occur. We have deliberately not included an RNase step in our experimental design, which would have destroyed this form of indirect interaction and would have focused the analysis on protein–protein complexes alone, because the aim was to get a comprehensive overview of the protein–protein interaction network, even if some of the interactions might be indirect and RNA mediated. A parallel analysis of the putative RNA components of the affinity isolated complexes was beyond the scope of this project.

### Characterization of Translation Initiation in Archaea

As mentioned in the Introduction, in recent years considerable progress has been obtained in the analysis of translation initiation in archaea. A very recent review gives an excellent overview of the progress (Schmitt et al., 2020). Breakthrough results were the structure determinations of preinitiation complexes from *P. abyssi* (Coureux et al., 2016, 2020). The complexes contained the ribosome, a short RNA, the initiation factors aIF1 and aIFA, and the ternary complex of the central factor aIF2 with the initiator tRNA and a GTP analog. Importantly for this study, the structures show the interaction of aIF1, aIF1A, and aIF2 of *P. abyssi* with one another and with the ribosome.

By far the highest number of studies on archaeal translation initiation have been performed with *Sulfolobus solfataricus*, which belongs to the kingdom of Crenarchaeota. Notably, it was found that the central heterotrimeric factor aIF2 in *Sulfolobus* does not only fulfill the homologous function of eIF2 to bring the initiator tRNA to the ribosome, but that the subunit aIF2γ has an additional role as stand-alone protein. It can bind to the 5'-end of transcripts and thereby it stabilizes transcripts and shields them from exonucleolytic degradation (Hasenöhrl et al., 2008). These results underscore the necessity to characterize archaeal translation initiation factors. Even if they are homologous to the eukaryotic factors, the two domains are separated in evolution by more than one billion of years, and the archaeal factors might have fewer or additional functions, different functions, or might even not be involved in translation initiation at all, despite their primary sequence similarities to eIFs.

In addition, the biological function of annotated aIFs might be different in different groups of archaea. The various phylogenetic groups of archaea are separated by billions of years, and it might well be that the functions of homologous proteins have evolved differently in different groups. This would be similar to bacteria, for which biodiversity in the mechanisms of translation initiation have become obvious (Malys and McCarthy, 2011).

### The Central Initiation Factor aIF2

The factor aIF2/eIF2 is present in archaea and eukaryotes, but it is not present in bacteria. It is a central factor because it guides the initiator tRNA to the P-site of the ribosome. In accordance with the crucial role of eIF2, all three subunits are essential in eukaryotes. Very unexpectedly, it was found that in *H. volcanii* only two subunits are essential (aIF2β and aIF2γ), while the gene for aIF2α could be deleted (Gäbel et al., 2013). This result indicated that either the complex is not heterotrimeric in haloarchaea, or that the aIF2βγ dimer lacking the native alpha subunit retains a residual function in translation initiation. In the present study, reciprocal co-isolation of all three subunits aIFα, aIFβ-2 and aIFγ was observed (Figure 3), showing that aIF2 is a heterotrimer also in *H. volcanii*, like in other archaea and eIF2 in eukaryotes. aIF2γ is the central and largest subunit (44.0kDa), while aIF2α and aIF2β-2 are somewhat smaller (29.5kDa, 22.2kDa). The structure of the preinitiation complex of *P. abyssi* revealed that aIF2γ is tightly bound to the 30S ribosomal subunit, while ribosomal binding of aIF2α and aIF2β is more loose (Schmitt et al., 2019). Therefore, it seems feasible that an aIF2βγ dimer retains the capability of binding the initiator tRNA, GTP, and the 30S subunit. In accordance with this view, in yeast it was shown that the alpha subunit contributed only slightly to the binding affinity of the initiator tRNA (Nika et al., 2001). However, initiator tRNA binding was found to be different in aIF2 from *S. solfataricus* and *P. abyssi* (Yatime et al., 2004; Schmitt et al., 2012; Naveau et al., 2013). In these archaeal systems the alpha subunit contributed to the tRNA binding, while the beta subunit had only a minor role. The contribution of the alpha and beta subunits to the tRNA binding of the haloarchaeal aIF2 is unclear. In any case, the alpha subunit is very important for the full function of aIF2 also in *H. volcanii*, because the deletion mutant has a severe growth defect under all tested conditions (Gäbel et al., 2013).

*Haloferax volcanii* contains two paralogs of the beta subunit, aIF2β-1 and aIF2β-2, in contrast to other archaea. They can functionally replace one another to some extent, because single deletion mutants of both genes could be constructed, while generation of a double deletion mutant was not possible (Gäbel et al., 2013). However, they are not equivalent. For example, a much higher number of proteins could be co-purified with aIF2β-2 than with aIF2β-1 (Figure 1B). In addition, only aIF2β-2 could be co-purified with both other subunits, not aIF2β-1 (Figure 3). However, aIF2β-1 could co-purify both other subunits, indicating that it is also part of a heterotrimeric aIF2 complex. The specific differential roles of the two aIF2β subunits remain to be determined.

aIF2β-1 and aIF2β-2 share an N-terminal region of about 130 amino acids (aa), and aIF2β-2 has an additional C-terminal domain of about 70 aa that is not present in aIF2β-1. A BLAST search with these extra 70 aa revealed that aIF2β-2 is widely distributed in halophilic and methanogenic archaea. However, it is also confined to these archaeal groups, so that the most plausible explanation is a gene duplication of an ancient version of aIF2 in the common ancestor of halophilic and methanogenic archaea, which was followed by the addition of 70 extra aa to only one of the two copies. The 70 aa have limited similarities to small proteins that are annotated as TRAM domain proteins. About 20years ago a bioinformatic analysis identified a conserved domain that occurred in two families of tRNA modifying enzymes, other proteins associated with translation, and a family of small, uncharacterized archaeal proteins and proposed the acronym TRAM (Anantharaman et al., 2001). Recently, it was shown that single domain small TRAM proteins in two psychrophilic methanogenic archaea have RNA-binding activity and are cold-shock proteins (Taha et al., 2016; Zhang et al., 2017). Deletion of a gene for a small TRAM domain protein in *Methanococcus maripaludis* reduced the growth rate and altered the levels of 55% of all transcripts (Li et al., 2019). Many 5'-UTRs were identified as potential targets of this protein, and three representative 5'-UTRs were unfolded by this TRAM protein *in vitro*. Taken together, it seems that after gene duplication one copy of the primordial aIF2β was fused with a small protein that added additional RNA-binding and RNA chaperone function and had a preference for 5'-UTRs.

To get a further insight into the evolution of a/eIF2β, a MSA was generated containing both paralogs from *H. volcanii* and selected proteins from haloarchaea, other archaea, and eukaryotes (Supplementary Figure S7). Obviously, the primordial protein had a size of about 130 aa and contained a CXXC and a CXXCG motif in its C-terminus. The four cysteines (red in Supplementary Figure S7) are highly conserved. They were shown to bind zinc (Gutiérrez et al., 2002), and the structures of several of these zinc fingers from several archaeal proteins have been solved (Gutiérrez et al., 2004; Nikonov et al., 2021). Later in evolution, eukaryotes have added an additional N-terminal domain of about 150 aa, which contains three poly-lysine stretches (blue in Supplementary Figure S7). These poly-lysine stretches are involved in mRNA-binding and the presence of at least on stretch is essential for function (Laurino et al., 1999; Salton et al., 2017). Taken together, a/eIF2β is an excellent example for protein evolution, in which the primordial protein of about 130 aa has been optimized by the addition of further RNA-binding domains either at the N-terminus (eukaryotes) or the C-terminus (halophilic and methanogenic archaea). This added functionality nicely explains the higher importance of aIF2β-2 compared to aIF2β-1 that was revealed in the present study.

In eukaryotes, eIF2α can be phosphorylated at a highly conserved serine residue. This is a key event in the integrated stress response of many or all eukaryotes, and it results in a downregulation of translation initiation (Kashiwagi et al., 2019; Marintchev and Ito, 2020). Phosphorylation of Ser51 (yeast numbering) of eIF2α inhibits the interaction of eIF2 with eIF2B, and, therefore, the GDP–GTP exchange is blocked and translation initiation stops (Gordiyenko et al., 2019). There are strong arguments that this regulatory step is not conserved in archaea, mainly (1) they do not contain homologs to the catalytic subunits of eIF2B, (2) there is no serine at the position homologous to yeast serine 51, and (3) the *Sulfolobus* aIF2 does not need an auxiliary factor for GDP/GTP exchange (Pedullà et al., 2005). In contrast to these arguments, it has been shown that aIF2α from *P. horikoshii* can be phosphorylated at a serine *in vitro* (Tahara et al., 2004). In archaeal aIF2 a serine is highly conserved at a position that is adjacent to the highly conserved serine in eukaryotes. We have exchanged this serine (serine 46 in *H. volcanii*) against alanine and aspartate to mimic the non-phosphorylated and the phosphorylated state (Schramm and Soppa, unpublished results). Both mutants grew nearly identical to the wildtype. In addition, the MS results after affinity isolation of aIF2α were searched, but a phosphorylated peptide could not be detected. These results indicate that regulation of translation initiation *via* phosphorylation of the conserved serine 46 of aIF2α does not occur in haloarchaea, in contrast to differential phosphorylation of the conserved serine 51 in eIF2α.

### The aIF Interaction Network With aIFs and With Other Proteins

In the present study, the protein–protein interaction network of translation initiation in *H. volcanii* could be unraveled (Figures 3–5 and Supplementary Tables S3–S6). A high number of interactions between aIFs as well as between aIFs and the ribosome could be detected. These results indicate that the bioinformatic annotation, which is based on protein sequence similarity to eukaryotic translation initiation factors, is correct, and that the aIFs are indeed involved in translation initiation in *H. volcanii*. Unexpectedly, also subunits of the RNA polymerase and other transcription proteins could be co-isolated with aIFs, indicating that translation and transcription are not independent in *H. volcanii* (discussed below). Two interaction hubs were identified in the aIF–aIF and the aIF–Rpo interaction networks, i.e., aIF5B and HVO_0966, which we propose to rename to aIF2Bα for reasons that are discussed in the next paragraph. The high importance of aIF5B was not unexpected, because it is one of only two universally conserved translation initiation factors (IF2 in bacteria and eIF5B in eukaryotes). It is involved in later stages of translation initiation and promotes binding of the large ribosomal subunit (Schmitt et al., 2020). Experimental evidence for this function also in archaea has been obtained with aIF5B from *Aeropyrum pernix* (Murakami et al., 2018). It should be noted that aIF5B binds to the initiator tRNA, like aIF2. Therefore, the co-purification strategy without an RNase step does not only include bait protein-mRNA-protein complexes (as discussed above), but might also include bait protein-tRNA-protein complexes.

Remarkably, the results with the second universally conserved initiation factor were totally different (aIF1A in archaea, eIF1A in eukaryotes, IF1 in bacteria). *H. volcanii* contains two paralogs, in contrast to other archaea, but none of them exhibited a high number of interactions to other aIFs or the ribosome. This is totally unexpected, because aIF1A is the third initiation factor that is part of the preinitiation complex of *P. abyssi*, in addition to aIF2 and aIF1 (Schmitt et al., 2019). It seems that either the affinities of the haloarchaeal aIF1As to the ribosome are not very high, or that the N-terminal hexahistidine tag interfered with complex formation. In any case, the two paralogs of aIF1A fulfill an essential role in *H. volcanii*, because it turned out to be impossible to delete both genes simultaneously (Gäbel et al., 2013).

### The Case of HVO_0966: aIF2Bα or Metabolic Enzyme

HVO_0966 turned out to be a very special case. It was included into the study, because it was annotated as a subunit of aIF2B. Later, the annotation was changed to the metabolic enzyme R15BI. There is experimental evidence that HVO_0966 homologs from *T. kodakarensis* and *P. horikoshii* function as R15BIs (Aono et al., 2012; Gogoi and Kanaujia, 2018). An enzyme kinetic characterization of the *T. kodakarensis* protein was performed, and a K_m_ value of 0.6mM for R15B was determined. The crystal structure of the *P. horikoshii* protein was determined, and R15B binding was verified. The enzyme R15BI is part of a three enzyme pathway that converts AMP into two molecules of 3-phosphoglycerate, and, thereby, funnels AMP into the central catabolic metabolism (Sato et al., 2007). The other two enzymes are AMP phosphorylase and ribulose-1,5-bisphosphate carboxylase (RuBisCO). Before this AMP salvage pathway was detected, the presence of RuBisCO in archaea had been an enigma, because RuBisCO is a central enzyme for CO_2_ fixation in the Calvin cycle, which is not present in archaea.

This experimental proof that the two homologs of HVO_0966 are metabolic enzymes strongly suggests that the haloarchaeal protein has the same function. On the other hand, our finding that HVO_0966 is one of only two interaction hubs in the translation initiation network and that it has a very high number of interactions with other aIFs, with the ribosome, and with Rpo subunits suggest evenly strongly that it functions as a translation initiation factor, and we propose to rename it to aIF2Bα. Additional experimental evidence in this direction is that HVO_0966 homologs from *P. horikoshii*, *P. furiosus*, and *T. acidophilum* bind to the cognate aIF2α as well as to the eIF2α from yeast (Dev et al., 2009). The structure of a protein annotated as aIF2Bα was solved and was used to model the structure of the eukaryotic regulatory subcomplex comprised of eIF2Bα, eIF2Bβ, and eIF2Bαδ (Kakuta et al., 2004). Taken together, strong experimental evidence in both directions has been provided.

The eukaryotic homologs of HVO_0966 form four subfamilies, three are comprised of the three regulatory subunits of eIF2B (eIF2Bα, eIF2Bβ, and eIF2Bδ), and the fourth subfamily consists of eukaryotic 5-methylthioribose-1-phosphate isomerase (MTPI). The phylogenetic analyses of selected archaeal and eukaryotic proteins confirmed the existence of these four eukaryotic subfamilies with very high bootstrap values (Figure 6, nodes A, B, C, D, and Supplementary Figures S4, 45). In contrast, the archaeal proteins did not form one monophyletic group. A few proteins group with the eukaryotic MTPI subfamily (node E), and indeed, it has been reported that the protein from *P. horikoshii* has this enzymatic activity (Gogoi and Kanaujia, 2018). The majority of the remaining archaeal proteins form one well-supported subfamily (node F). The annotation is very mixed; however, the group includes the two proteins that were shown to have the R15BI activity as well as HVO_0966 from *H. volcanii*, which is an interaction hub in the translation initiation network. Two explanations for this contradiction seem possible, (1) the archaeal proteins have evolved into the two different functions in the long time the species were separated, or (2) the archaeal proteins have both functions and are in fact moonlighting proteins.

So-called moonlighting proteins can fulfill at least two very different functions in different biological processes. A classic example is enolase, which is part of the glycolytic pathway, but which is also involved in RNA degradation (Henderson and Martin, 2013). However, the number of known moonlighting proteins has increased drastically in recent years (Jeffery, 2020; Liu and Jeffery, 2020; Singh and Bhalla, 2020; Beaufay et al., 2021; Rodríguez-Saavedra et al., 2021; Turek and Irving, 2021). An indication that the archaeal proteins might have two different functions is the recent observation that the eukaryotic eIF2Bα still has the ability to bind sugar phosphates with high affinity, e.g., fructose-6-phosphate has a K_D_ of 9.4μM (Hao et al., 2021). The binding of sugar phosphate enhances the formation of the complete eIF2B decamer. The authors speculated that the high-affinity sugar binding couples the nutrient status of the cell to the translation rate. This would lead to an automatic downregulation of translation, a very costly process, when nutrients become scarce.

It could be envisaged that the archaeal proteins of the subfamily node F represent a primordial state, in which sugar metabolism is coupled to translation regulation not only *via* the sole binding of sugar phosphates, but in which they have a second role as enzymes. A first example of a moonlighting translation initiation factor has been reported, because aIF5A from *S. solfataricus* exhibits also the enzymatic function of a ribonuclease (Bassani et al., 2019). Future experiments are needed to unravel whether the archaeal enzymes indeed has two different functions. Until then we propose to rename HVO_0966 to aIF2Bα because of its central position in the translation initiation protein interaction network.

### The Rpo–Rpo Interaction Network

Structure and function of archaeal RNA polymerases (Rpo) and their relationship to eukaryotic RNA polymerases have been studied very intensely (Grohmann and Werner, 2011; Werner and Grohmann, 2011; Nagy et al., 2015; Fouqueau et al., 2018). They are composed of catalytic subunits, an assembly platform, a stalk, and auxiliary subunits. The co-isolation of several Rpo subunits with aIFs prompted us to extend our study to co-isolation experiments with Rpo subunits. In total, seven subunits from different parts of the complex were chosen. With only one exception reciprocal co-isolation of Rpo subunits was observed (Figure 7). Additional subunits were also co-isolated, that were not used as bait proteins. In total, 10 Rpo subunits could be co-purified, which strongly indicates that the whole enzyme complex could be co-purified when a single subunit was tagged and used a bait protein. Therefore, the approach was well suited to unravel the RNA polymerase interaction network.

### Transcription-Translation Coupling in Archaea and in *E. coli*

In contrast to eukaryotes, in which transcription and translation occur in two different cellular compartments, the nucleus and the cytoplasm, in prokaryotes both processes occur in the cytoplasm. Therefore, translation can begin before a gene has been fully transcribed. This allows the coupling of transcription and translation. A regulatory process called attenuation has already been described decades ago (Yanofsky, 1981). In this case, the speed of the first translating ribosome determines whether the gene is fully transcribed, or whether a transcription termination stemloop is formed.

Very recently, a much more direct coupling between transcription and translation in *E. coli* has been reported. In 2017 two structures of a complex of RNA polymerase with the 30S ribosomal subunit were solved (Demo et al., 2017; Kohler et al., 2017). Since then, several additional structures were obtained, and the current opinion is that not a single interaction complex exists, but that complex formation between the RNA polymerase and the ribosome can be very dynamic, and that the interaction can be either direct or mediated by the protein NusG, which can bind to the ribosome as well as to Rpo. Several reviews summarize the current knowledge (Artsimovitch, 2018; Conn et al., 2019; Irastortza-Olaziregi and Amster-Choder, 2020; Wang et al., 2020). However, transcription–translation coupling is not universal in bacteria, for example, it has been shown that it is not coupled in *Bacillus subtilis* and probably other gram-positive bacteria (Wang and Artsimovitch, 2021).

In contrast to the rapidly- growing experimental evidence for *E. coli* and other bacteria, nearly nothing is known about the coupling of transcription and translation in archaea. Electron microscopic observation of lysed cells of *T. kodakarensis* revealed that polysomes were very close to dispersed strands of genomic DNA, and, based on this observation, it was proposed that transcription and translation are coupled in this species (French et al., 2007). In this Research topic of Frontiers of Microbiology a theoretical paper was published that proposed transcription–translation coupling to occur in archaea based on the universal conservation of NusG/Spt5 and NusA, which connect Rpo and the ribosome in *E. coli* (Weixlbaumer et al., 2021). NusG and NusA are also encoded in the genome of *H. volcanii*. We searched for the two proteins in the MS results, but neither of them was co-isolated with any of the seven Rpo subunits used as bait proteins.

However, the reciprocal co-isolation of aIFs and Rpo subunits as well as the co-isolation of 21 ribosomal proteins with Rpo subunits as bait proteins provides very strong evidence that translation and transcription are not independent in *H. volcanii*, but the two processes are coupled (Figure 8 and Supplementary Tables S9 and S10). To our knowledge this is the second experimental study that indicates that transcription and translation are coupled in archaea. Clearly, more analyses with more archaeal species are highly needed for a better understanding of the molecular mechanism and the distribution in the domain of archaea.

## Conclusion

In a very comprehensive approach the protein–protein interaction networks of translation initiation and the RNA polymerase of *H. volcanii* have been elucidated. Manifold aIF-aIF, aIF-ribosome, aIF-Rpo, and aIF-transcription factor interactions were observed. Two proteins turned out to be interaction hubs, i.e., the universally conserved factor aIF5B as well as HVO_0966, which we propose to rename aIF2Bα. The reciprocal co-isolation of aIFs and Rpo subunits as well as Rpo subunits and ribosomal proteins gives an additional evidence that transcription and translation are coupled in archaea.

## Data Availability Statement

The protein interactions from this publication have been submitted to the IMEx (http://www.imexconsortium.org) consortium through IntAct (Orchard et al., 2014) and assigned the identifier IM-29121. The original Excel files with the MS results have been deposited at a German repository called “Hessenbox” and can be downloaded from the following site: https://hessenbox-a10.rz.uni-frankfurt.de/getlink/fiMwRZN2jZQYjQfyWzLk7QsB/Raw%20MS%20Data%20Schramm.zip. The mass spectrometry proteomics data have been deposited in the ProteomeXchange Consortium vie the PRIDE partner repository (Perez-Riverol et al., 2019) with the dataset identifier PXD028666.

## Author Contributions

FS and JS designed the experiments. FS performed all molecular biological experiments and co-affinity purifications. UL performed all peptide mass fingerprinting and MS/MS experiments. FS, AB, UL, and JS analyzed the data. FS and AB generated all tables, figures and supplementary data. JS wrote a first draft of the manuscript. All authors contributed to the final version and approved it for publication.

## Funding

This project was funded by the German Research Foundation (Deutsche Forschungsgemeinschaft, DFG) through the grants So264/24 and So264/29 to JS.

## Conflict of Interest

The authors declare that the research was conducted in the absence of any commercial or financial relationships that could be construed as a potential conflict of interest.

## Publisher’s Note

All claims expressed in this article are solely those of the authors and do not necessarily represent those of their affiliated organizations, or those of the publisher, the editors and the reviewers. Any product that may be evaluated in this article, or claim that may be made by its manufacturer, is not guaranteed or endorsed by the publisher.

## Footnotes

^1^ https://www.ebi.ac.uk/Tools/sss/ncbiblast/

^2^ https://scied.com/pr_cmbas.htm

^3^ https://www.ebi.ac.uk/
