# Supplementary material for: Elucidation of the Translation Initiation Factor Interaction Network of Haloferax volcanii Reveals Coupling of Transcription and Translation in Haloarchaea
Source: Front Microbiol. 2021 Oct 26;12:742806. doi: 10.3389/fmicb.2021.742806 (PMC8576121; doi:10.3389/fmicb.2021.742806)
Supplement: Supplementary file 1 [file Data_Sheet_1.PDF]

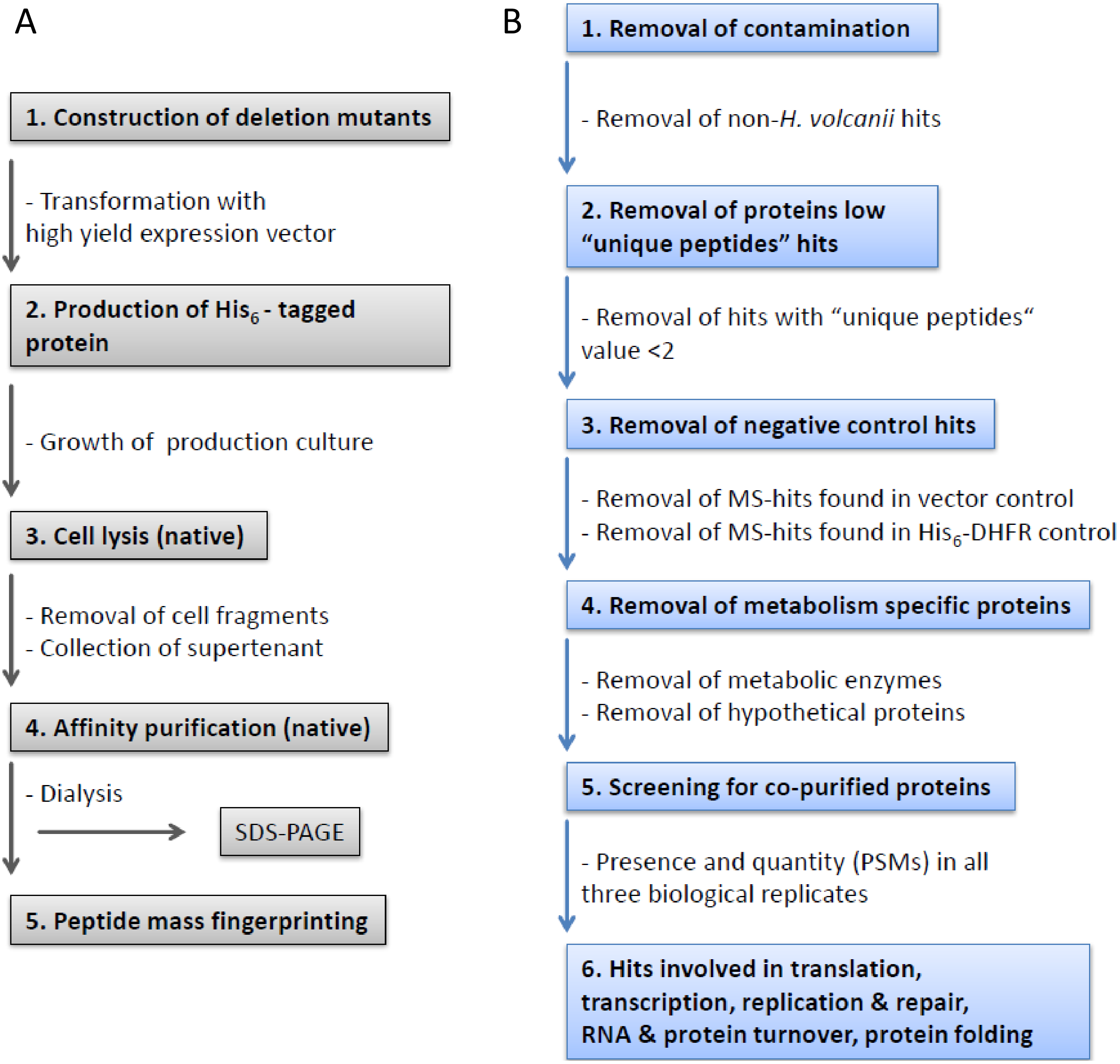

Supplementary Figure 1. **Overwiew of the experimental and bioinformatic workflow.**
A) Schematic Overview of the experimental workflow of cell harvest and co-affinity isolation. **B**) Schematic Overview of the bioinformatic workflow used for the identification of co-purified proteins. All steps are shown that have been applied to process the raw data received after LC-MS to identify the co-purified proteins. The Peptide Spectrum Matches (PSMs) served as a semi-quantitative indicator.

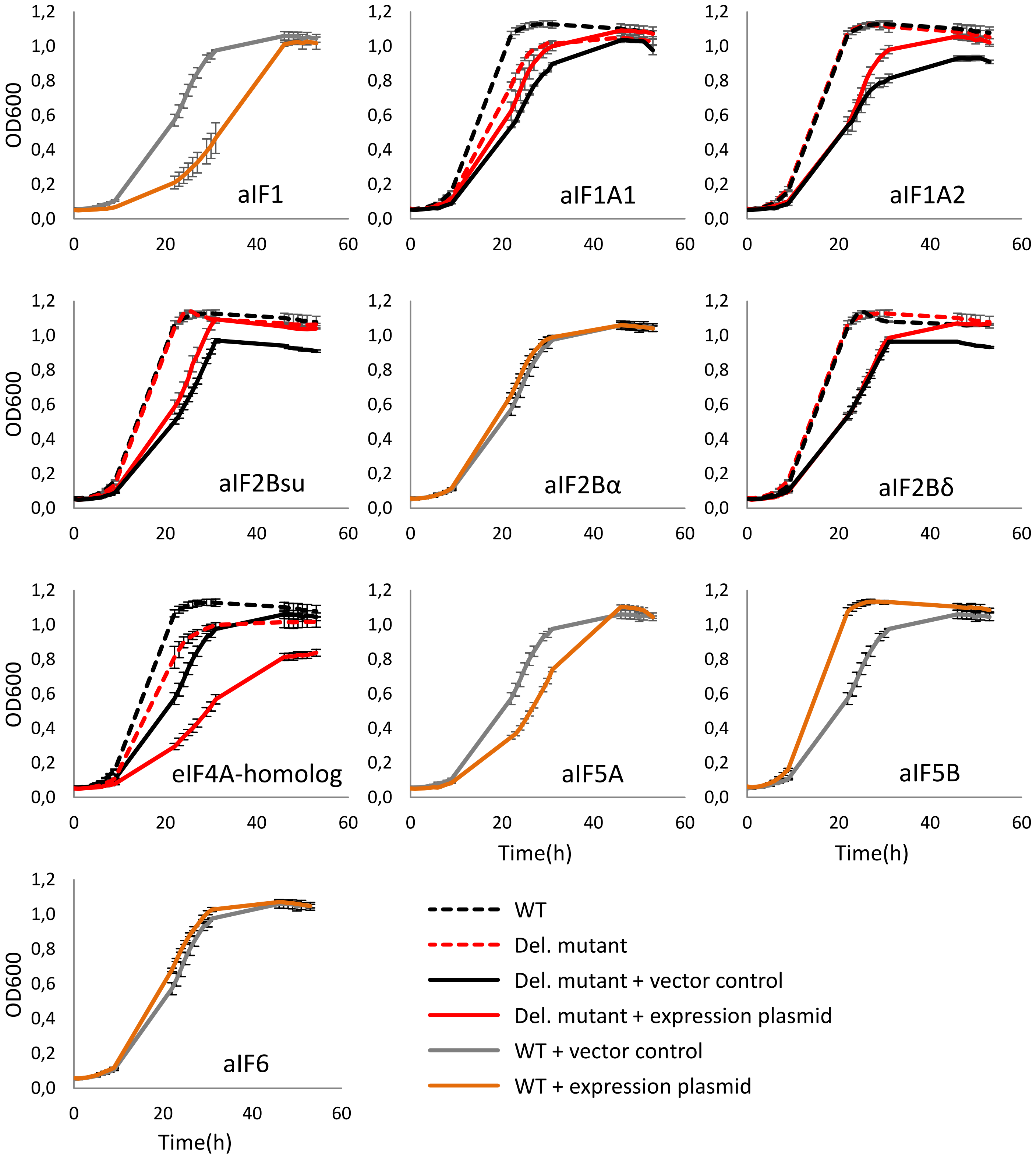

Supplementary Figure 2. **Growth curves of aIF deletion mutants and overproduction strains.** All cells were grown in triplicates under optimal growth conditions in complex media (all vector-containing cultures in the presence of Novobiocin) and in 96-well plates. OD600 was measured frequently, and average values and their standard deviations are shown. Dotted lines – plasmid-free cultures, solid lines – plasmid-containing cultures, red lines – deletion mutants, black lines – wildtype, grey line – wildtype with empty vector, orange line – wildtype with expression plasmid.

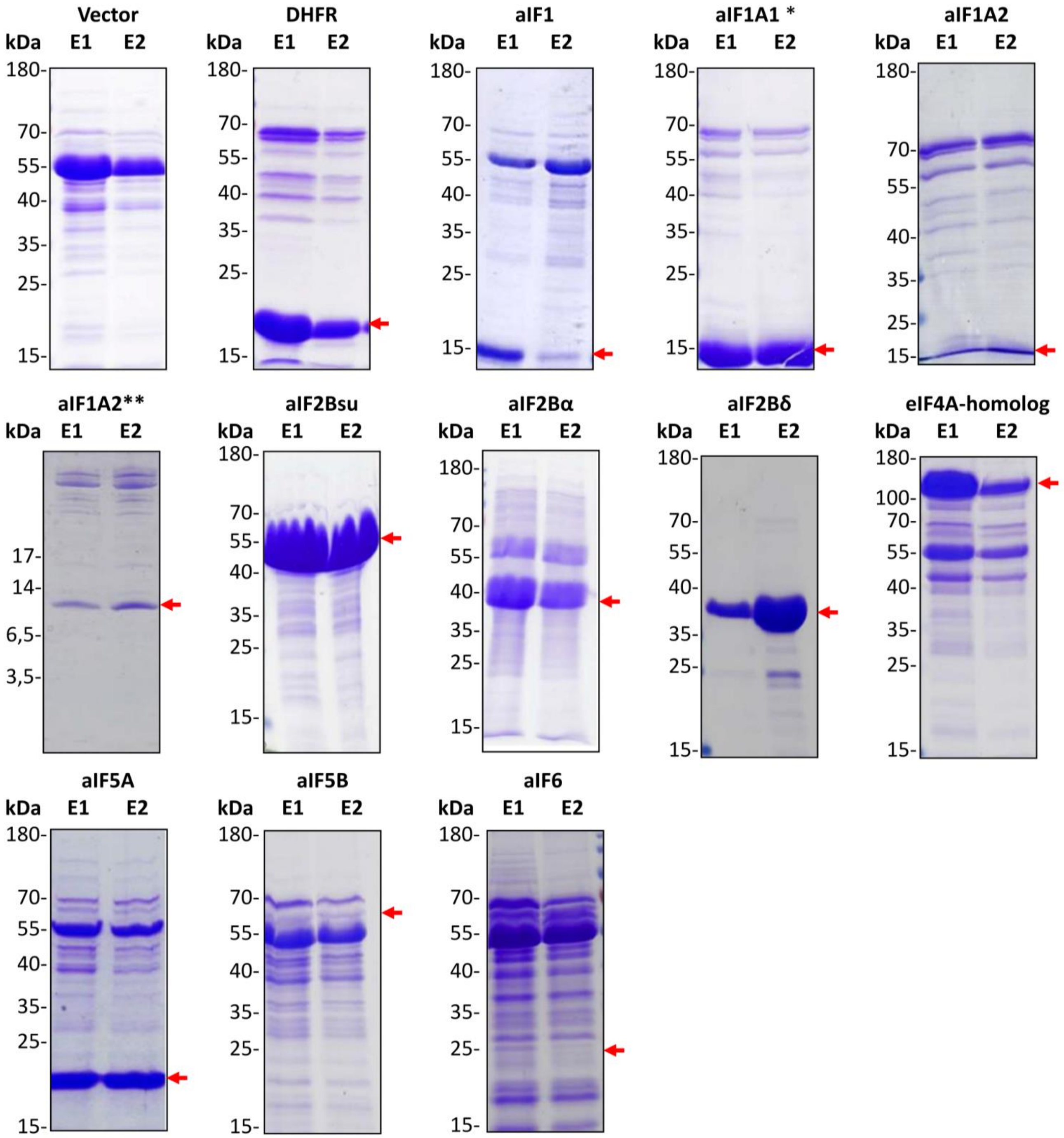

Supplementary Figure 3. **SDS-Page of the elution fractions 1 and 2 of His$_6$-tagged aIFs.** The red arrow indicates the overexpressed His$_6$-tagged bait protein. Examples given are representative for three biological replicates.

*The aIF1A1 overexpression construct contained the native 83 nucleotides long 3'-UTR after the ORF. Without the UTR, aIF1A1 was not detectable after overexpression.

** Here, a Tricine-based gel was used for better visibility of the aIF1A2 protein.

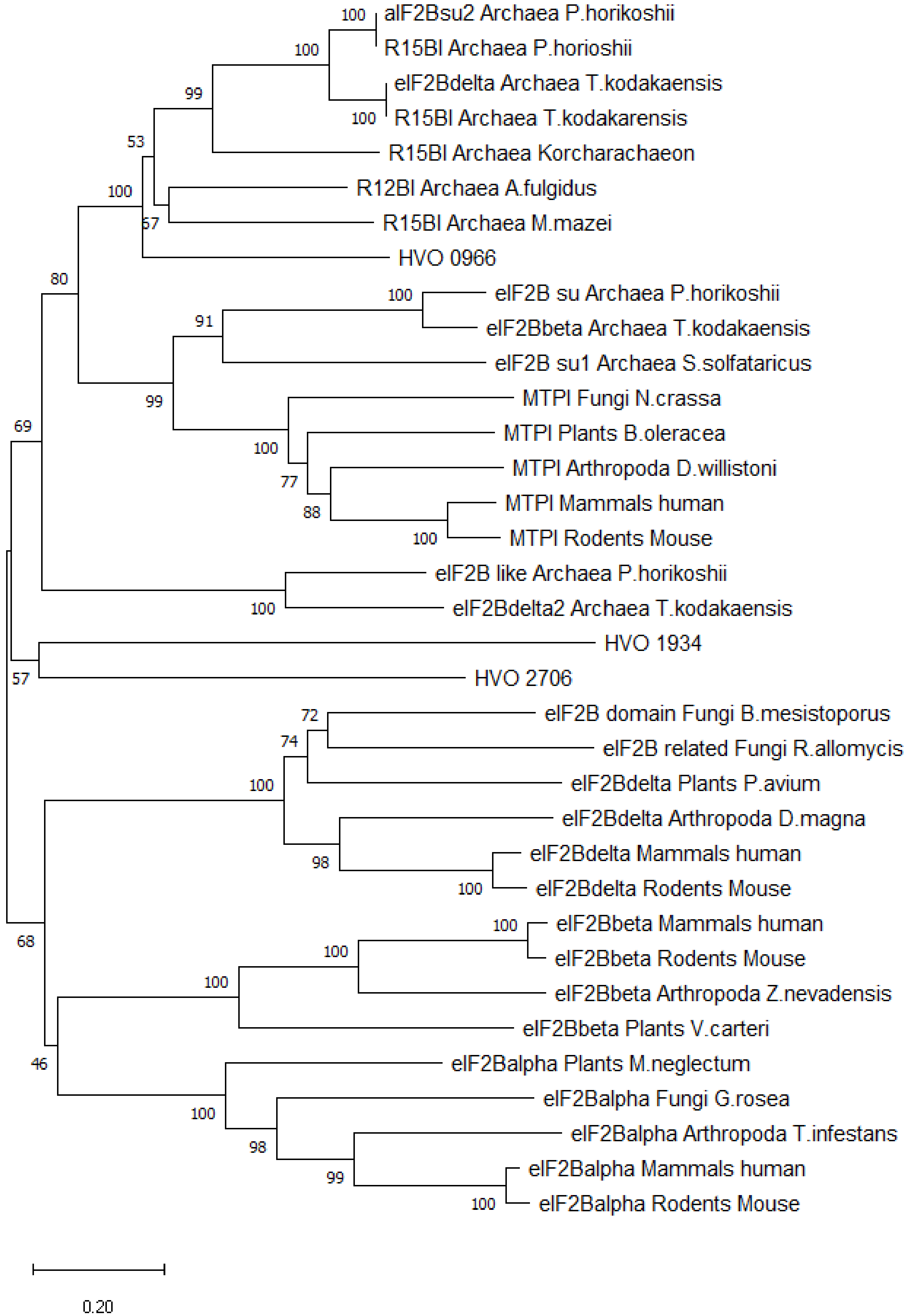

Supplementary Figure 4. **Neighbor Joining (NJ) tree with 1000 bootstrap replications**. The bootstrap values (%) are shown at each node.

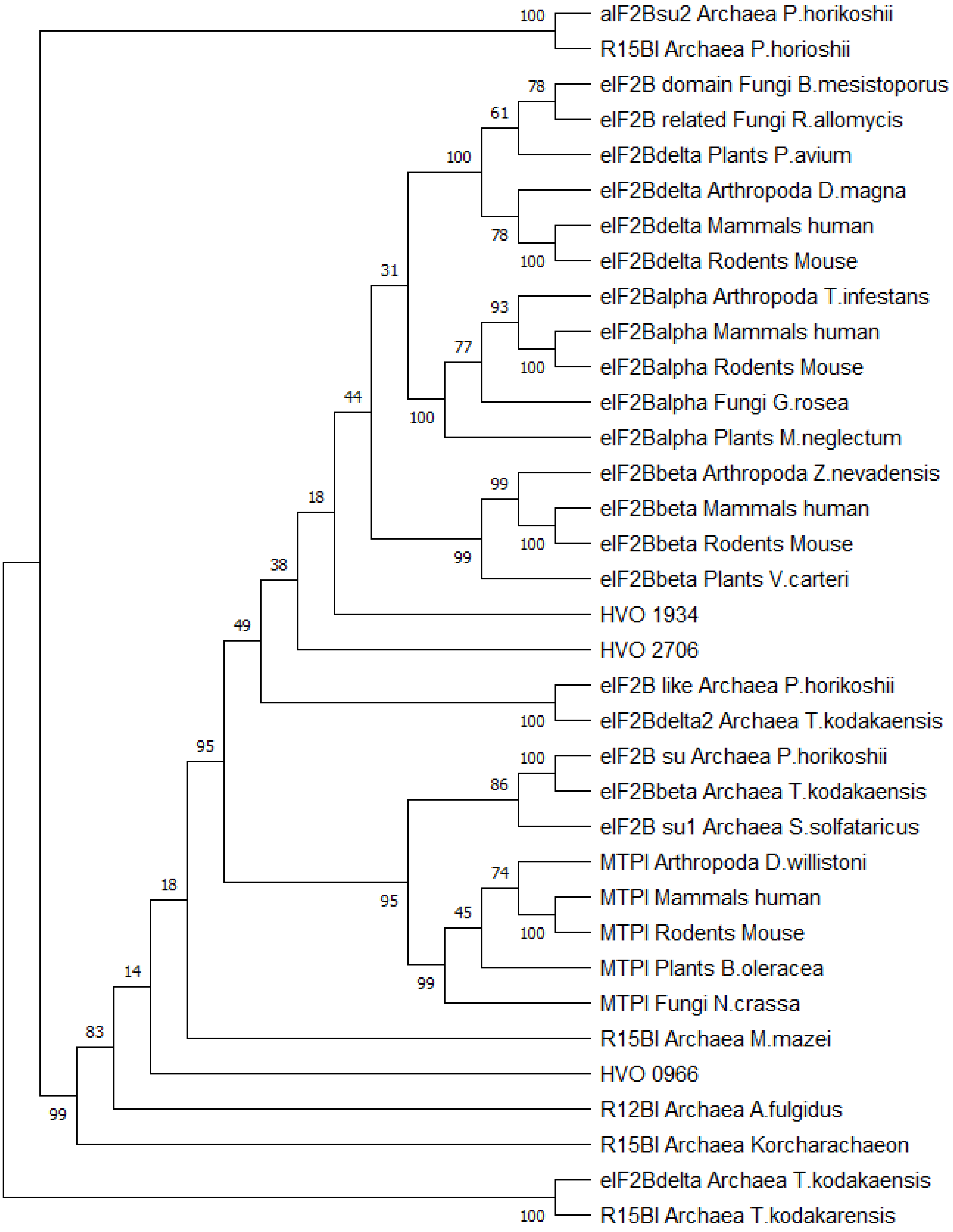

Supplementary Figure 5. Maximal Parsimony (MP) tree with 1000 bootstrap replications. The bootstrap values (%) are shown at each node.

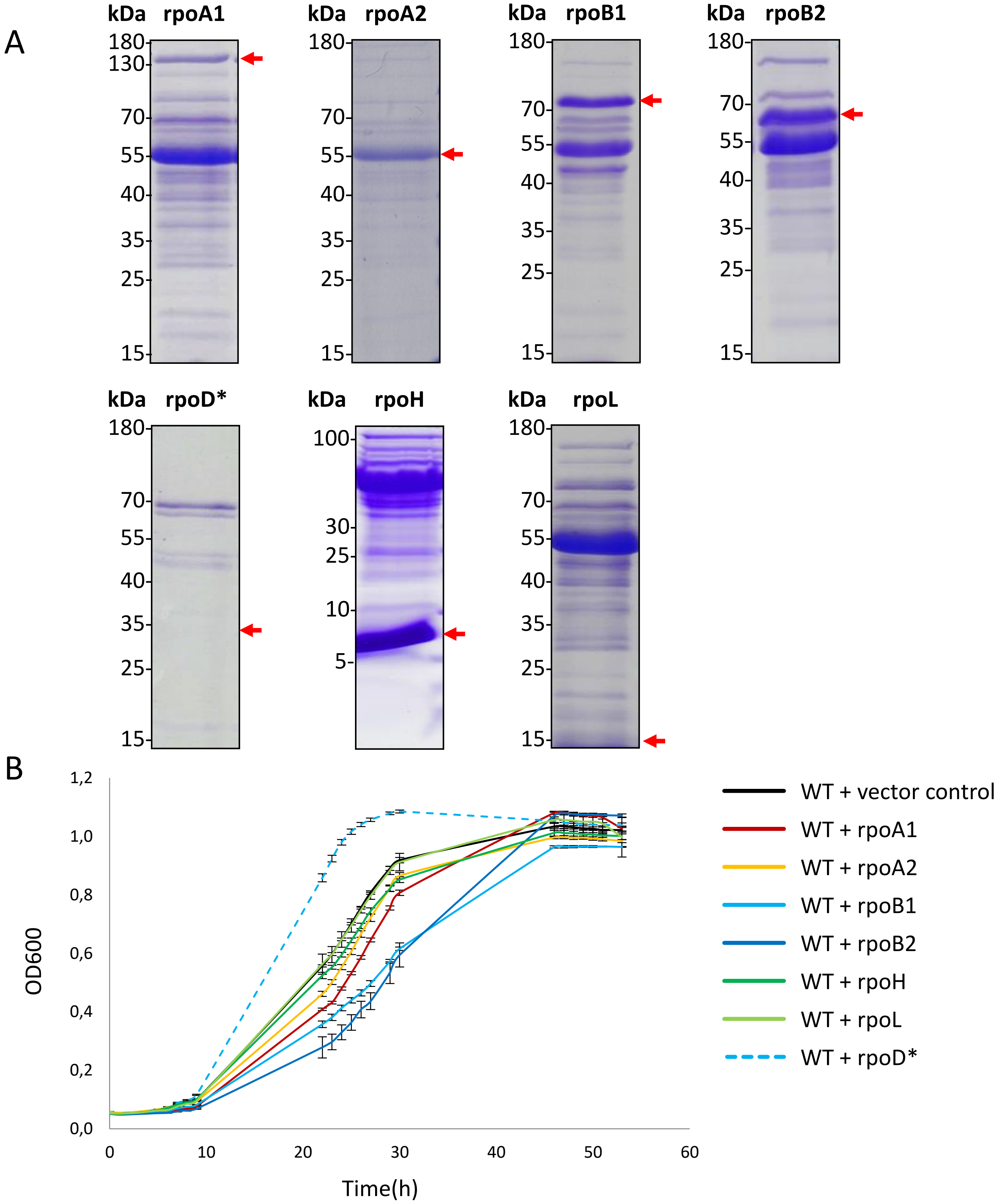

Supplementary Figure 6. Overproduction of His$_6$-tagged RNA polymerase subunits.
A) SDS-Page of the elution fraction 2 of indicated Rpo subunits. The red arrow indicates the overexpressed His$_6$-tagged bait protein. Examples given are representative for three biological replicates. **B)** Growth curves of cultures of the wildtype and the strains overproducing the indicated Rpo subunit**.** All cultures were grown in triplicates under optimal growth conditions in complex media (all vector-containing cultures in the presence of Novobiocin) and 96-well plates. OD600 was measured frequently, and average values and their standard deviations are shown.
* RpoD yield after overproduction was very low.

```
Sac_cerevisiae                MSSDLAAELGFDPALKKKKKTKKVIPDDFDAAVNGK------------------------     36
Dro_melanogaster              ----MDAEDGFDPTLLKKKKKKKTT-FDLDAALGLEDDTK------KEDP--QDE-----     42
Homo_sapiens                  ---MSGDEMIFDPTMSKKKKKKKKP-FMLDEEGDTQTEETQPSETKEVEPEPTEDKDLEA     56
Rattus_norwegicus             ---MSGDEMIFDPTMSKKKKKKKKP-FMLDEEGDAQTEETQPSETKEVEPEPAEEKDVEA     56
Sul._solfataricus             ------------------------------------------------------------     0
Met._jannaschii               ------------------------------------------------------------     0
Mbc._thermoautotrophicum      ------------------------------------------------------------     0
Pco._furiosus                 ------------------------------------------------------------     0
Hfx._volcanii_1               ------------------------------------------------------------     0
Hfx._volcanii_2               ------------------------------------------------------------     0
Hqu._walsbii                  ------------------------------------------------------------     0
Nal_madadii                   ------------------------------------------------------------     0
Hco_morrhuae                  ------------------------------------------------------------     0
Hru_sodomense                 ------------------------------------------------------------     0

Sac_cerevisiae                ------ENGS---GD--DLFAGLKKKKKKSKSVSA----------------DAEAEKEPT     69
Dro_melanogaster              ---ASAEGGAAAEEDNLDLESFGKKKKKKKKPFNMDEIEAAIPSFGGDDVAASEEPEEEE     99
Homo_sapiens                  DEEDTRKKDASDDLDDLNFFNQKKKKKKTKKIFDIDEAEEGVKDLKIES--DVQEPTEPE     114
Rattus_norwegicus             DEEDSRKKDASDDLDDLNFFNQKKKKKKTKKIFDIDEAEEAIKDVKIES--DAQEPAEPE     114
Sul._solfataricus             ------------------------------------------------------------     0
Met._jannaschii               ------------------------------------------------------------     0
Mbc._thermoautotrophicum      ------------------------------------------------------------     0
Pco._furiosus                 ------------------------------------------------------------     0
Hfx._volcanii_1               ------------------------------------------------------------     0
Hfx._volcanii_2               ------------------------------------------------------------     0
Hqu._walsbii                  ------------------------------------------------------------     0
Nal_madadii                   ------------------------------------------------------------     0
Hco_morrhuae                  ------------------------------------------------------------     0
Hru_sodomense                 ------------------------------------------------------------     0

Sac_cerevisiae                DDIAEALGELSLKKKKKKTKDSSVDAFEKELAKAGLDNVDAESKEGT----PSANSSIQQ     125
Dro_melanogaster              INLDMDFSMAKKKKKKSKK-KE-LDELFADQ--------ADDDKSEDKENEDNSSTWFGS     149
Homo_sapiens                  DDLDIMLGNKKKKKKNVKFPD-EDEILEKDEA---LEDEDNKKDDGISFSNQTGPAWAGS     170
Rattus_norwegicus             DDLDIMLGNKKKKKKNVKFPD-EDEILEKDEA---LEDEDSKKDDGISFSNQTGPAWAGS     170
Sul._solfataricus             -----------------------------------------------------------M     1
Met._jannaschii               ------------------------------------------------------MSDLEN     6
Mbc._thermoautotrophicum      ------------------------------------------------------------     0
Pco._furiosus                 ----------------------------------------------------------ME     2
Hfx._volcanii_1               ------------------------------------------------------------     0
Hfx._volcanii_2               ------------------------------------------------------------     0
Hqu._walsbii                  ------------------------------------------------------------     0
Nal_madadii                   ------------------------------------------------------------     0
Hco_morrhuae                  ------------------------------------------------------------     0
Hru_sodomense                 ------------------------------------------------------------     0

Sac_cerevisiae                EVGLPYSELLSRFFNILRTNNPELAGDRSGPKFRIPPPVCLR-DGKKTIFSNIQDIAEKL     184
Dro_melanogaster              DRDYTYDELLKRVFEIILDKNPDMAAGRK-PKFVMRPPQVLRVGTKKTSFANFMDIAKTL     208
Homo_sapiens                  ERDYTYEELLNRVFNIMREKNPDMVAGEK-RKFVMKPPQVVRVGTKKTSFVNFTDICKLL     229
Rattus_norwegicus             ERDYTYEELLNRVFNIMREKNPDMVAGEK-RKFVMKPPQVVRVGTKKTSFVNFTDICKLL     229
Sul._solfataricus             SSEKEYVEMLDRLYSKL----PEKGR-KE-GTQSLPNMIILN-IGNTTIIRNFAEYCDRI     54
Met._jannaschii               IDYYDYKALLKRARSQI----PDYVF-QK-DRFELPEIEILI-EGNRTIIRNFRELAKAV     59
Mbc._thermoautotrophicum      --MDDYEKLLERAIDQL----PPEVF-ET-KRFEVPKAYSVI-QGNRTFIQNFREVADAL     51
Pco._furiosus                 IDYYDYEKLLEKAYQEL----PENVKHHK-SRFEVPGALVTI-EGNKTIIENFKDIADAL     56
Hfx._volcanii_1               ---MDYDDQLDRALSAS----PDVAE-GD-DRFSVPEPTVRQ-EGNVTVYENFAATHDRL     50
Hfx._volcanii_2               ---MEYQTALDRALNVL----PERNV-EQ-ERLTVPDPSGET-DGAFTRLTNLGEIADAL     50
Hqu._walsbii                  ---MDYTESLNRALDEL----PERRD-EE-SRLSIPDPVGET-DGAFTRLTNIGEIADAL     50
Nal_madadii                   ---MDYESSLDRAMEDV----PDIGG-DE-ERLQIPDAQTQK-DGAFTRFTNLGEIADVL     50
Hco_morrhuae                  ---MDYEASLDRAMDSV----PDLDT-GD-SRLDVPDAEAQK-DGAFTRLTNLGNVADAL     50
Hru_sodomense                 ---MDYESSLDRAMDEV----PDLGG-SD-ERLSVPDPEWQK-DGAFTRLTNLSAIADAL     50
                                   *   *.:  .      *            :            *   *:    . :
```

```
Sac_cerevisiae                HRSPEHLIQYLFAELGTSGSVDGQKRLVIKGKFQSKQMENVLRRYILEYVTCKTCKSINT    244
Dro_melanogaster              HRLPKHLLDFLLAELGTSGSMDGNQQLIIKGRFQPKQIENVLRRYIKEYVTCHTCRSPET    268
Homo_sapiens                  HRQPKHLLAFLLAELGTSGSIDGNNQLVIKGRFQQKQIENVLRRYIKEYVTCHTCRSPDT    289
Rattus_norwegicus             HRQPKHLLAFLLAELGTSGSIDGNNQLVIKGRFQQKQIENVLRRYIKEYVTCHTCRSPDT    289
Sul._solfataricus             RREDKICMKYLLKELAAPGNVDDKGELVIQGKFSSQVINTLMERFLKAYVECSTCKSLDT    114
Met._jannaschii               NRDEEFFAKYLLKETGSAGNLEG-GRLILQRRISPELLKSRINDFLREYVICRECGKPDT    118
Mbc._thermoautotrophicum      NRDPQHLLKFLLRELGTAGNLEG-GRAILQGKFTHFLINERIEDYVNKFVICHECNRPDT    110
Pco._furiosus                 NRDPQHLLKFLLREIATAGTLEG-RRVVLQGRFTPYLIANKLKKYIKEYVICPVCGSPDT    115
Hfx._volcanii_1               AREATHVLKFFQTELGTSAQIDDRGRARFTGEFRQRRIADALDEYVESFVLCSECGSPDT    110
Hfx._volcanii_2               SRTPAHLHSAIQRTLGTSGQLEG-DRARYSGSFSINDFEEAIDGYVEEYVICSECGLPDT    109
Hqu._walsbii                  GRPAEHVHRNVQGELGTNGQLED-ERARYNGSFSVADFEAAIDEYVIEYVTCTECGLPDT    109
Nal_madadii                   SREDEHLHRFVQREMGTSGKFEE-GRGRYNGTFSEQDFNAAVDAYVDEYVLCTECGLPDT    109
Hco_morrhuae                  SRDPEHLHRVIQRDLGTNGQFSG-DRARYNGTFSGEDFDAAIDSYSQEFVICSECGLPDT    109
Hru_sodomense                 SRDPEHVHSKIQQELGTAGQYED-GRARYSGNFRERDFQAAIDSYIESFVTCSECGLPDT    109
                               *        .    .: .  .   .      :    :   :  :   :* *  *   :*

Sac_cerevisiae                ELKREQSNRLFFMVCKSCGSTRSVSSIKTGFQATVGKRRRM-------------------    285
Dro_melanogaster              ILQK--DTRLFFLQCESCGSRCSVASIKSGFQAVTGKRAAIRAKTT--------------    312
Homo_sapiens                  ILQK--DTRLYFLQCETCHSRCSVASIKTGFQAVTGKRAQLRAKAN--------------    333
Rattus_norwegicus             ILQK--DTRLYFLQCETCHSRCSVASIKTGFQAVTGKRAQLRAKAN--------------    333
Sul._solfataricus             ILKK--EKKSWYIVCLACGAQTPVKPL---------------------------------    139
Met._jannaschii               KIIK--EGRVHLLKCMACGAIRPIRMI---------------------------------    143
Mbc._thermoautotrophicum      RIIR--EGRISLLKCEACGAKAPLKNV---------------------------------    135
Pco._furiosus                 KIIK--RDRFHFLKCEACGAETPIQHL---------------------------------    140
Hfx._volcanii_1               KLTS--QRGADVLKCDACGALSAIPDL---------------------------------    135
Hfx._volcanii_2               RLVT--EDGVDMLRCEACGAFRPVQKRSSVSNK-RQREAVEEGRTYEVEITGTGRKGDGV    166
Hqu._walsbii                  RLTT--ENGVDMLRCEACGAFRPVQKQTTTQQQSQSGPAVEEGATYEVEITGTGRKGDGV    167
Nal_madadii                   RLVR--EDRTPMLRCDACGAFRPVTKRSTSAAQQQQQDAVEEGKTYTVEITGTGRKGDGV    167
Hco_morrhuae                  RLEM--EGRTQMLRCEACGAFRPVEKNTGSQET-EQRPDVEEGRTYEVKITGTGRKGDGV    166
Hru_sodomense                 RLET--ENRTPMLRCEACGAFRPVAKQNTSNTQ-RQEEAVESGTTYELEIVGTGRKGDGV    166
                               :          : * :* :   :

Sac_cerevisiae                ------------------------------------- 285
Dro_melanogaster              ------------------------------------- 312
Homo_sapiens                  ------------------------------------- 333
Rattus_norwegicus             ------------------------------------- 333
Sul._solfataricus             ------------------------------------- 139
Met._jannaschii               ------------------------------------- 143
Mbc._thermoautotrophicum      ------------------------------------- 135
Pco._furiosus                 ------------------------------------- 140
Hfx._volcanii_1               ------------------------------------- 135
Hfx._volcanii_2               AQRGKYTIFVPGAQEGQTVRIYIKNTSGSLAFARLA- 202
Hqu._walsbii                  AEKGKFTIFVSGAEEGQTVRVQIERTSGTLAFAQVI- 203
Nal_madadii                   AEKGEYTIFVPGAQEGDVVDIYIKNISGNLAFARLD- 203
Hco_morrhuae                  AEQGKYTIFVPGAQEGDVVDIYIENISGTLAFARLA- 202
Hru_sodomense                 AERGEYTIFVPGAQEGETVTAYIKNVSGNLAFARRED 203
```

Supplementary Figure 7. Multiple sequence alignment of a/eIF2β translation initiation factors from selected archaea and eukaryotes, representing various phylogenetic lineages. The alignment was generated using Clustal Omega at the website of the European Bioinformatics Institute (www.ebi.ac.uk). Positions of increasing degrees of conservation are marked with ., :, and *. The four concerved cysteines in two CXXC motifs, which form a zinc finger, are shown in red. The three poly-lysine stretches confined to eukaryotic proteins are shown in blue. The C-terminal extensions that are found exclusively in halophilic and methanogenic archaea are shown in purple.

Supplementary Table 1. Primers for generating and/or confirming *H.Volcanii* deletion mutants

Name	Sequence
HVO_0136 P1	CCGACGCGCCGCCGACGCTGT
HVO_0136 P2	GCGGAGCTGGTCGGCAAGGTCTCGGCGGCTCTCGTT
HVO_0136 P3	AGCCGCCGAGACCTTGCCGACCAGCTCCGCCGCGAA
HVO_0136 P4	GACGACCTGTCCCTTGTCGTG
HVO_0359 P1	GGTTGAGGGAATCCGCAACG
HVO_0359 P2	GCATCGTTATCGCTCGCTCATTGGTGTGGTCACGC
HVO_0359 P3	ACCACACCAATGAGCGAGCGATAACGATGCAACAGGC
HVO_0359 P4	GTTCCGTCGGGCGATTCTG
HVO_0569 P1	CAGCCGCTCAGTGGATGTGC
HVO_0569 P2	GACGGCGAACTCGTCGGCGACGTTCCGTACCATGC
HVO_0569 P3	GTACGGAACGTCGCCGACGAGTTCGCCGTCTGAGC
HVO_0569 P4	CGCTCCCGGCGATGTACCAC
HVO_0699 P1	ACACCAAAACCCACCACAAGGTCGATATCTATG
HVO_0699 P2	TTCGCGGTGGTACTCGAGTTCGCCGGGGTCAGGCCA
HVO_0699 P3	GACCCCGGCGAACTCGAGTACCACCGCGAACGCAGC
HVO_0699 P4	GGGTCAGCTCGGCGACGCCC
HVO_1333 P1	ACGCGGGCGAGTCGCTGCC
HVO_1333 P2	GTCAGTCCCAGAAGCCGTCTGGCATCGCCCGAGGTTACG
HVO_1333 P3	ATGCCAGACGGCTTCTGGGACTGACGGGGTGGATTCCGGTT
HVO_1333 P4	GTCGCTCGGCTCCGACATCCTCG
HVO_1678 P1	TTCTCGGTTCAGTCGGCTTGCGG
HVO_1678 P2	TCGTTTTTACAGGTAGTCCATGCGCGGCAGTACG
HVO_1678 P3	GCGCATGGACTACCTGTAAAAACGAACGTCCGGCGG
HVO_1678 P4	CGGTCGGCCTCGCCGCCGCCG
HVO_1934 P1	CAGCTGCGAGACAGCGCGACGACG
HVO_1934 P2	GTCGTCCCACGCGCGACGTGTGGCATATCGTGACTTACGGC
HVO_1934 P3	GCCACACGTCGCGCGTGGGACGACTGAGTCGGGAGTGC
HVO_1934 P4	CGGTTTCGGGGCTCCGGTCGAGG
HVO_2242 P1	CGTGATTTCGTTCGGCAGCCCG
HVO_2242 P2	GACCGATTCAGGCCATAGGGGTTCGAGTGTCTGGC
HVO_2242 P3	CGAACCCCTATGGCCTGAATCGGTCGCACGCGTCG
HVO_2242 P4	CGGCTACAAGGCGCTCGACTCC
HVO_2706 P1	CCCATCTGCGAGGCGGGCCAGC
HVO_2706 P2	TCAGAACTCGATGACGGTCTCGTCTATCATACGAGAACGTCGCCGTATCG
HVO_2706 P3	ATGATAGACGAGACC GTCATCGAGTTCTGAGCGGGCGGCG
HVO_2706 P4	TCGGTTCCTCGTCAGTGACCGCGCC
HVO_A0637 P1	GTCCGCTGTTCTGGGAGACGACGGTAACCG
HVO_A0637 P2	TTACTGAATGTGGCCTTCATTTGACGAGGTCATCCACGATTCCCCTACAACAC
HVO_A0637 P3	ATGACCTCGTCAAATGAAGGCCACATTCAGTAATCATTTTCTCCGAAAGGAGAG
HVO_A0637 P4	GGGAACCAAGACGGCCGACGACGC
HVO_B0053 P1	GACGCTGGCCGACCGCTATC
HVO_B0053 P2	GGCGTCGTGAAGCGTTCGGAGCGCTTCGAGTTCTGC
HVO_B0053 P3	CTCGAAGCGCTCCGAACGCTTCACGACGCCTTCCC
HVO_B0053 P4	ACGACGCTCCGCCAGACTC
Probe 0359 for	TCAACAAGATGGACGTCGTC
Probe 0359 rev	ACTTCCTCGTGGTGCATCTC
Probe 0569 for	CGTTCGCCCAGCGAGACACC
Probe 0569 rev	GCCGCGTAGGCGTCGGCCAT
Probe 0966 for	CACGAACGGGGGCGCACCGGTC
Probe 0966 rev	GCGGTCGTCCATGCGAGAACTACGGG
Probe 1333 for	ACCCTTCACCCGCTCTCGAACGGCGTAGC
Probe 1333 rev	GCCGTCTGGCATCGCCCGAGGTTACG
Probe 1934 for	GCGTGGGACGACTGAGTCGGGAGTGC

**Probe 1934 rev**	CGGTTTCGGGGCTCCGGTCGAGG
**Probe 2242 for**	GGACGCGCTCTCGCGGACGC
**Probe 2242 rev**	CTGCGCGCCGGGCACGAAGA
**Probe 2706 for**	CCCATCTGCGAGGCGGGCCAGC
**Probe 2706 rev**	ATACGAGAACGTCGCCGTATCG
**Probe A0637 for**	ACCCAACGACGAACCGGCGA
**Probe A0637 rev**	CACGTGCGGTACTTCATGCG
**Probe B0053 for**	TGAACATCACCGGAACGACC
**Probe B0053 rev**	CTCCGAGGTCGTCGAGCAGC

Supplementary Table 2. Primers for generating expression vectors with His_6-tagged gene variants.

Name	Sequence
Oligo-His-Tag_for	CATGGGCCACCACCACCACCACCACGC
Oligo-His-Tag_rev	CATGGCGTGGTGGTGGTGGTGGTGGCC
NcoI_His_0117_for	TGATGCCCATGGGCCACCACCACCACCACCACGCCACTTCGCGCCTCCTT
KpnI_0117 rev	GAATTGGGTACCTCAGTCGATGTAATCGAG
HVO_0136_His_NheI_for	GATGCCCATGGGCCACCACCACCACCACCACGCTAGCGGCATGAGCGACGACGAGAACGAG
HVO_0136_KpnI_rev	CGAATTGGGTACCGCTTACTGGATGTGACCTTCG
NcoIH0136mit3UTRKpnI_for	GATGCCCATGGGCCACCACCACCACCACCACGCCAGCGACGACGAGAACGAGAG
NcoIH0136mit3UTRKpnI_rev	GAATTGGGTACCGGCGAGATGAAAAGGGCACAGG
HVO_0699_His_NheI_for	GATGCCCATGGGCCACCACCACCACCACCACGCTAGCGGCATGAAGTACAGCGGATGGCCT
HVO_0699_KpnI_rev	CGAATTGGGTACCGTTACTCTTCGTCGCCGCTGC
NcoI_His_0966_KpnI_for	GATGCCCATGGGCCACCACCACCACCACCACGCTAGCGGCATGGACGACCGCGTA
NcoI_His_0966_KpnI_rev	CGAATTGGGTACCGCTACGGTTCGGCCCGCGGC
NcoI_1333_KpnI_for	GATGCCCATGGGCCACCACCACCACCACCACGCTAGCGGCATGCCAGACGGCGAGGCCGC
NcoI_1333_KpnI_rev	CGAATTGGGTACCGTCAGTCCCAGAACGACTGG
HVO_1678_His_NheI_for	GATGCCCATGGGCCACCACCACCACCACCACGCTAGCGGCATGGACTACGACGACCAACTG
HVO_1678_KpnI_rev	CGAATTGGGTACCGTTACAGATCGGGAATCGCTG
NdeI_His_1901_for	ACTAGGACCATATGCACCACCACCACCACCACGTGACGAAAAACCCACAA
1901_KpnI_rev	GGCGAATTGGGTACCTCACTTGAGCGTCCC
NcoI_His_1934_KpnI_for	GATGCCCATGGGCCACCACCACCACCACCACGCTAGCGGCATGCCACACGTCGCCACCGT
NcoI_His_1934_KpnI_rev	CGAATTGGGTACCGTCAGTCGTCCCACGCCGCC
NcoI_His_1946_KpnI_for	TGATGCCCATGGGCCACCACCACCACCACCACGCTAGCGGCATGTCGGAAGTCTGCTCGA
NcoI_His_1946_KpnI_rev	CGAATTGGGTACCGTCACGCGACGTTGAACCCC
NheI_1963_KpnI_for	ACCACCACGCTAGCGGCATGTCCGACACTGATT
NheI_1963_KpnI_rev	CGAATTGGGTACCGCTATTTGCCCCAGAAGGGG
HVO_2242_His_NheI_for	GATGCCCATGGGCCACCACCACCACCACCACGCTAGCGAGTATCAGACTGCGCTCGACCGA
HVO_2242_KpnI_rev	CGAATTGGGTACCTCAGGCGAGTCGCGCGAACGC
NcoI_His_2300_KpnI_for	GATGCCCATGGGCCACCACCACCACCACCACGCTAGCGGCATGGCGAAAGAGCAGAAGCA
NcoI_His_2300_KpnI_rev	CGAATTGGGTACCGTTAGACGATCTTTCGCTGG
NcoI_His_2706_KpnI_for	GATGCCCATGGGCCACCACCACCACCACCACGCTAGCGGCATGATAGACGAGACCATCGA
NcoI_His_2706_KpnI_rev	CGAATTGGGTACCGTCAGAACTCGATGACGCCC
NcoIHis_A0637_KpnI_for	GATGCCCATGGGCCACCACCACCACCACCACGCCAGTGAAAATCAAGGGCGTCG
NcoIHis_A0637_KpnI_rev	CGAATTGGGTACCTTACTGAATGTGGCCTTCGCGCCGAA
NcoI_H0346_KpnI_for	GATGCCCATGGGCCACCACCACCACCACCACGCTAGCGGCGTAGACGTAAGCCAACACAA
NcoI_H0346_KpnI_rev	CGAATTGGGTACCGTCATTCAATCACCAGTCGGTATACGACT
NcoI_His_0347_KpnI_for	GATGCCCATGGGCCACCACCACCACCACCACGCTAGCGGCATGAACCGACAAGCG
NcoI_His_0347_KpnI_rev	CGAATTGGGTACCGTTAGTCGTCCGCGGGTGCG
NcoI_H0348_KpnI_for	GATGCCCATGGGCCACCACCACCACCACCACGCTAGCGGCGCTCAGGCACAGCGAGA
NcoI_H0348_KpnI_rev	CGAATTGGGTACCGTTAGACCGCGTCCTTCAGTTCG
NcoI_His_0349_KpnI_for	GATGCCCATGGGCCACCACCACCACCACCACGCTAGCGGCATGTCAATGCAGACA
NcoI_His_0349_KpnI_rev	CGAATTGGGTACCGTCAGTCATCGGATTCGACC
NcoI_His_0350_KpnI_for	GATGCCCATGGGCCACCACCACCACCACCACGCTAGCGGCATGACTGATACCGAC
NcoI_His_0350_KpnI_rev	CGAATTGGGTACCGTCAGTCGTCGGACGGTTCG
NcoI_H1042_KpnI_for	GATGCCCATGGGCCACCACCACCACCACCACGCTAGCGGCGAACTGCGGGTCATCGA
NcoI_H1042_KpnI_rev	CGAATTGGGTACCGTTACGCGTCGATACCGGCGCGG
NcoI_H2781_KpnI_for	GATGCCCATGGGCCACCACCACCACCACCACGCTAGCGGCGTAAACGACTTCCAGGTCGA
NcoI_H2781_KpnI_rev	CGAATTGGGTACCGTTTAAACTTACAGTGCGACCTTCTCTTC

Supplementary Table 3. Co-isolated proteins with aIF2 subunits.

						Bait			
MW [kDa]	Group	Specification	Genloci	Accession	Description	aIF2α	aIF2β-1	aIF2β-2	aIF2γ
29.5	Translation	aIFs	HVO_0699	D4GT46	**aIF2α**	bait	+	+	+
14.9	Translation	aIFs	HVO_1678	D4GZP2	**aIF2β-1**		bait		
22.2	Translation	aIFs	HVO_2242	L9VAS4	**aIF2β-2**	+		bait	+
43.9	Translation	aIFs	HVO_1901	D4GTD4	**aIF2γ**	+	+	+	bait
34.9	Translation	aIFs	HVO_0966	L9USK7	**aIF2Bα**	+			+
65.3	Translation	aIFs	HVO_1963	D4GTJ2	**aIF5B**	+		+	+
45.6	Translation	aEFs	HVO_2413	L9V6J4	**aEF1α**	+		+	+
57.8	Translation	aEFs	HVO_2575	L9V605	**aEF1α-like protein**	+			
80.4	Translation	aEFs	HVO_0356	L9UK07	**aEF2**	+		+	+
24.8	Translation	30S rpS	HVO_1145	D4GWA5	**30S rpS3Ae**			+	
23.3	Translation	30S rpS	HVO_2544	D4GTX6	**30S rpS5**	+			
13.4	Translation	30S rpS	HVO_2782	L9V5R1	**30S rpS11**			+	
23.1	Translation	50S rpL	HVO_2757	P41199	**50S rpL1**	+			
25.6	Translation	50S rpL	HVO_2561	D4GTZ3	**50S rpL2**			+	+
37.2	Translation	50S rpL	HVO_2564	L9V5K9	**50S rpL3**			+	
27	Translation	50S rpL	HVO_2563	D4GTZ5	**50S rpL4**			+	
36.9	Translation	50S rpL	HVO_2756	P41198	**50S rpL10**	+			
19.8	Translation	50S rpL	HVO_0484	D4GS24	**50S rpL10e**	+			
22.5	Translation	50S rpL	HVO_0561	L9UPT6	**50S rpL15e**	+			
20.2	Translation	50S rpL	HVO_2545	P50563	**50S rpL18**			+	
12.7	Translation	50S rpL	HVO_2779	D4GWY3	**50S rpL18e**			+	
16.9	Translation	50S rpL	HVO_2559	L9V5K3	**50S rpL22**			+	
17.1	Translation	50S rpL	HVO_2543	L9V5X1	**50S rpL30**	+		+	
81.2	Translation	transferase	HVO_2736	D4GW73	**tRNA(Met) cytidine acetyltransferase TmcA**	+			
108.8	Transcription	RNAP-subunits	HVO_0349	D4GZX6	**rpoA1**	+			
46.1	Transcription	RNAP-subunits	HVO_0350	D4GZX7	**rpoA2**	+			
58.9	Transcription	RNAP-subunits	HVO_0347	L9UK99	**rpoB2**	+			+
11.7	Transcription	regulator	HVO_1133	D4GW85	**HTH domain protein**				+
23.8	Transcription	regulator	HVO_A0121	D4GQF3	**HTH-10 family transcription regulator**	+			
28	Transcription	regulator	HVO_2108	D4GUQ5	**IclR family transcription regulator**	+	+	+	
18.1	Transcription	regulator	HVO_1792	D4GSB6	**Lrp/AsnC family transcription regulator**	+			
8.3	Transcription	regulator	HVO_2723	D4GW47	**RNA-binding protein Lsm**	+			
19.9	Transcription	regulator	HVO_1727	L9VGC6	**TATA-box-binding protein**	+			
19.8	Transcription	regulator	HVO_0163	L9UFW5	**Transcriptional regulator**	+			
28	Transcription	regulator	HVO_0179	L9UGU5	**Transcriptional regulator**				+
27.5	Transcription	regulator	HVO_2928	L9UK32	**Transcriptional regulator**	+	+		
30.9	Transcription	regulator	HVO_2636	L9V878	**Transcriptional regulator**	+			
15.2	Protein folding	chaperone	HVO_0450	D4GRZ1	**Hsp20-type molecular chaperone**	+			
37.3	Replication and Repair	repair	HVO_0104	Q48328	**DNA repair and recombination protein RadA**	+			
24.6	Replication and Repair	repair	HVO_2383	L9V6G6	**DNA repair and recombination protein RadB**	+			
69.6	Replication and Repair	replication	HVO_0415	D4GRV6	**Repair helicase UvrD**	+			
34.6	Replication and Repair	replication	HVO_0292	L9UJU8	**Replication factor A**	+			
87.4	Replication and Repair	replication	HVO_1571	L9VEG4	**Type 2 DNA topoisomerase 6 subunit B**	+			
23.2	RNA turnover	ribonuclease	HVO_2438	L9V6L9	**Ribonuclease H I**	+			
50.1	RNA turnover	ribonuclease	HVO_2724	L9V5P6	**Ribonuclease J**			+	
59.3	Protein turnover	thermosome	HVO_0455	O30560	**Thermosome subunit 2**	+		+	
55.2	Protein turnover	thermosome	HVO_0778	Q9HHA2	**Thermosome subunit 3**	+		+	

Supplementary Table 4. The aIF-aIF interaction network.

				Bait													
MW [kDa]	Genloci	Accession	Description	aIF1	aIF1A-1	aIF1A-2	aIF2α	aIF2β-1	aIF2β-2	aIF2γ	aIF2Bsu	aIF2Bα	aIF2Bδ	eIF4A-hom.	aIF5A	aIF5B	aIF6
10.8	HVO_1946	D4GTH5	**aIF1**	bait													
11.5	HVO_0136	D4GZ79	**aIF1A1**		bait												
11.2	HVO_A0637	D4GRU5	**aIF1A2**	+		bait											
29.5	HVO_0699	D4GT46	**aIF2α**				bait	+	+	+		+			+		
14.9	HVO_1678	D4GZP2	**aIF2β-1**					bait									
22.2	HVO_2242	L9VAS4	**aIF2β-2**				+		bait	+		+					
43.9	HVO_1901	D4GTD4	**aIF2γ**				+	+	+	bait		+					
43.2	HVO_1934	D4GTG3	**aIF2Bsu**								bait						
34.9	HVO_0966	L9USK7	**aIF2Bα**	+	+	+	+			+	+	Bait		+	+	+	+
30.7	HVO_2706	L9V7F9	**aIF2Bδ**									+	bait				
105	HVO_1333	D4GXK1	**eIF4A-homolog**											bait			
14.2	HVO_2300	L9V7A1	**aIF5A**												bait		
65.3	HVO_1963	D4GTJ2	**aIF5B**	+	+	+	+		+	+	+	+		+	+	bait	+
23	HVO_0117	D4GYW3	**aIF6**														bait

Supplementary Table 5. Overview of ribosomal proteins co-isolated with aIFs.

				Bait													
**MW [kDa]**	**Genloci**	**Accession**	**Description**	**aIF1**	**aIF1A-1**	**aIF1A-2**	**aIF2α**	**aIF2-β1**	**aIF2-β2**	**aIF2-γ**	**aIF2Bsu**	**aIF2Bα**	**aIF2Bδ**	**eIF4A-hom**	**aIF5A**	**aIF5B**	**aIF6**
28.3	HVO_2773	L9V5L7	**30S ribosomal protein S2**									+					
33.4	HVO_2558	D4GTZ0	**30S ribosomal protein S3**	+								+		+			
24.8	HVO_1145	D4GWA5	**30S ribosomal protein S3Ae**						+			+					
26.6	HVO_2552	L9V7Y2	**30S ribosomal protein S4e**	+								+		+	+		
23.3	HVO_2544	D4GTX6	**30S ribosomal protein S5**				+							+			
22.6	HVO_0354	L9UJR0	**30S ribosomal protein S7**	+								+		+			
11.5	HVO_0360	L9ULL7	**30S ribosomal protein S10**	+												+	
13.4	HVO_2782	L9V5R1	**30S ribosomal protein S11**						+			+					
18.9	HVO_2784	L9V5M6	**30S ribosomal protein S13**	+								+					
17.5	HVO_1148	L9UVI2	**30S ribosomal protein S15**									+					
12.7	HVO_2555	D4GTY7	**30S ribosomal protein S17**									+					
16	HVO_2560	L9V659	**30S ribosomal protein S19P**	+	+							+					
11.6	HVO_1896	D4GTC9	**30S ribosomal protein S24e**	+													
23.1	HVO_2757	P41199	**50S ribosomal protein L1**	+			+				+	+	+	+	+	+	+
25.6	HVO_2561	D4GTZ3	**50S ribosomal protein L2**	+	+				+	+	+	+		+		+	+
37.2	HVO_2564	L9V5K9	**50S ribosomal protein L3**	+					+			+		+	+		+
27	HVO_2563	D4GTZ5	**50S ribosomal protein L4**	+	+				+			+		+			+
19.6	HVO_2548	L9V5X7	**50S ribosomal protein L6**									+		+			
36.9	HVO_2756	P41198	**50S ribosomal protein L10**	+			+					+		+			
19.8	HVO_0484	D4GS24	**50S ribosomal protein L10e**				+										
16	HVO_2778	D4GWY1	**50S ribosomal protein L13**	+													
22.5	HVO_0561	L9UPT6	**50S ribosomal protein L15e**				+										
20.2	HVO_2545	P50563	**50S ribosomal protein L18**						+			+					
12.7	HVO_2779	D4GWY3	**50S ribosomal protein L18e**	+					+			+					
16.9	HVO_2559	L9V5K3	**50S ribosomal protein L22**	+					+		+	+		+		+	
13.4	HVO_2553	L9V5Y5	**50S ribosomal protein L24**		+												
17.1	HVO_2543	L9V5X1	**50S ribosomal protein L30**	+	+		+		+		+	+		+		+	
25.8	HVO_2547	D4GTX9	**50S ribosomal protein L32e**	+													

Supplementary Table 6. All (non-metabolic) proteins co-purified with aIFs.

					Bait													
MW [kDa]	Group	Genloci	Accession	Description	aIF1	aIF1A-1	aIF1A-2	aIF2α	aIF2β-1	aIF2β-2	aIF2γ	aIF2Bsu	aIF2Bα	aIF2Bδ	eIF4A-hom	aIF5A	aIF5B	aIF6
10.8	Translation	HVO_1946	D4GTH5	aIF1	bait													
11.5	Translation	HVO_0136	D4GZ79	aIF1A-1		bait												
11.2	Translation	HVO_A0637	D4GRU5	aIF1A-2	+		bait											
29.5	Translation	HVO_0699	D4GT46	aIF2α				bait	+	+	+		+			+		
14.9	Translation	HVO_1678	D4GZP2	aIF2β-1					bait									
22.2	Translation	HVO_2242	L9VAS4	aIF2β-2				+		bait	+		+					
43.9	Translation	HVO_1901	D4GTD4	aIF2γ				+	+	+	bait		+					
43.2	Translation	HVO_1934	D4GTG3	aIF2Bsu								bait						
34.9	Translation	HVO_0966	L9USK7	aIF2Bα	+	+	+	+			+	+	bait		+	+	+	+
30.7	Translation	HVO_2706	L9V7F9	aIF2Bδ									+	bait				
104.5	Translation	HVO_1333	D4GXK1	eIF4A-hom.											bait			
14.2	Translation	HVO_2300	L9V7A1	aIF5A												bait		
65.3	Translation	HVO_1963	D4GTJ2	aIF5B	+	+	+	+		+	+	+	+		+	+	bait	+
23	Translation	HVO_0117	L9UI67	aIF6														bait
45.6	Translation	HVO_2413	L9V6J4	aEF1α	+	+	+	+		+	+	+	+		+	+	+	+
57.8	Translation	HVO_2575	L9V605	aEF1α-like protein	+			+				+			+	+	+	
80.4	Translation	HVO_0356	L9UK07	aEF2	+	+		+		+	+	+	+		+	+	+	+
28.3	Translation	HVO_2773	L9V5L7	30S rpS2									+					
33.4	Translation	HVO_2558	D4GTZ0	30S rpS3	+								+		+			
24.8	Translation	HVO_1145	D4GWA5	30S rpS3Ae						+			+					
26.6	Translation	HVO_2552	L9V7Y2	30S rpS4e	+								+		+	+		
23.3	Translation	HVO_2544	D4GTX6	30S rpS5				+							+			
22.6	Translation	HVO_0354	L9UJR0	30S rpS7	+								+		+			
11.5	Translation	HVO_0360	L9ULL7	30S rpS10	+												+	
13.4	Translation	HVO_2782	L9V5R1	30S rpS11						+			+					
18.9	Translation	HVO_2784	L9V5M6	30S rpS13	+								+					
17.5	Translation	HVO_1148	L9UVI2	30S rpS15									+					
12.7	Translation	HVO_2555	D4GTY7	30S rpS17									+					
16	Translation	HVO_2560	L9V659	30S rpS19P	+	+							+					
11.6	Translation	HVO_1896	D4GTC9	30S rpS24e	+													
23.1	Translation	HVO_2757	P41199	50S rpL1	+			+				+	+	+	+	+	+	+
25.6	Translation	HVO_2561	D4GTZ3	50S rpL2	+	+				+	+	+	+		+		+	+
37.2	Translation	HVO_2564	L9V5K9	50S rpL3	+					+			+		+	+		+
27	Translation	HVO_2563	D4GTZ5	50S rpL4	+	+				+			+		+			+
19.6	Translation	HVO_2548	L9V5X7	50S rpL6									+		+			
36.9	Translation	HVO_2756	P41198	50S rpL10	+			+					+		+			
19.8	Translation	HVO_0484	D4GS24	50S rpL10e				+										
16	Translation	HVO_2778	D4GWY1	50S rpL13	+													
22.5	Translation	HVO_0561	L9UPT6	50S rpL15e				+										
20.2	Translation	HVO_2545	P50563	50S rpL18						+			+					
12.7	Translation	HVO_2779	D4GWY3	50S rpL18e	+					+			+					
16.9	Translation	HVO_2559	L9V5K3	50S rpL22	+					+		+	+		+		+	
13.4	Translation	HVO_2553	L9V5Y5	50S rpL24		+												
17.1	Translation	HVO_2543	L9V5X1	50S rpL30	+	+		+		+		+	+		+		+	
25.8	Translation	HVO_2547	D4GTX9	50S rpL32e	+													
81.2	Translation	HVO_2736	D4GW73	tRNA(Met) cytidine acetyltransferase TmcA	+			+							+			
40.5	Translation	HVO_0911	D4GUZ0	GTP-binding protein Drg									+					

					Bait													
MW [kDa]	Group	Genloci	Accession	Description	aIF1	aIF1A-1	aIF1A-2	aIF2α	aIF2β-1	aIF2β-2	aIF2γ	aIF2Bsu	aIF2Bα	aIF2Bδ	eIF4A-hom	aIF5A	aIF5B	aIF6
108.8	Transcription	HVO_0349	D4GZX6	rpoA1	+	+		+					+		+	+	+	
46.1	Transcription	HVO_0350	D4GZX7	rpoA2	+			+					+		+			
67.7	Transcription	HVO_0348	L9UJM2	rpoB1	+													
58.9	Transcription	HVO_0347	L9UK99	rpoB2	+			+			+		+		+	+	+	
28.1	Transcription	HVO_2781	L9V5W2	rpoD	+								+					
13.6	Transcription	HVO_2748	D4GWA3	rpoF									+					
8.5	Transcription	HVO_0346	D4GZX3	rpoH									+					
10.4	Transcription	HVO_1042	D4GVL8	rpoL	+													
16.9	Transcription	HVO_2029	L9VBJ7	AsnC family transcriptional regulator		+							+					
13.5	Transcription	HVO_A0135	D4GQG4	HTH domain protein	+													
11.7	Transcription	HVO_1133	D4GW85	HTH domain protein	+	+					+	+	+		+			
23.4	Transcription	HVO_1695	D4H040	HTH domain protein	+	+									+		+	
23.8	Transcription	HVO_A0121	D4GQF3	HTH-10 family transcription regulator	+			+							+		+	
28	Transcription	HVO_1501	D4GYE7	HTH-type transcriptional regulator GlpR	+												+	
28.4	Transcription	HVO_B0114	D4GPB5	IclR family transcription regulator	+								+		+		+	+
28.1	Transcription	HVO_B0201	D4GPK2	IclR family transcription regulator													+	
27.9	Transcription	HVO_A0583	D4GRP3	IclR family transcription regulator	+										+		+	
28	Transcription	HVO_2108	D4GUQ5	IclR family transcription regulator	+	+	+	+	+	+		+	+		+	+	+	+
18.5	Transcription	HVO_B0066	D4GP68	Lrp/AsnC family transcription regulator	+												+	
18.1	Transcription	HVO_1792	D4GSB6	Lrp/AsnC family transcription regulator	+		+	+				+	+		+		+	+
16.9	Transcription	HVO_0240	D4GZI5	Lrp/AsnC family transcription regulator									+					
11.4	Transcription	HVO_2869	D4GXH6	PadR family transcription regulator	+							+			+			
13.6	Transcription	HVO_0730	D4GT98	PadR family transcription regulator RosR									+					
13.1	Transcription	HVO_1090	D4GW09	Putative transcription factor									+					
8.3	Transcription	HVO_2723	D4GW47	RNA-binding protein Lsm	+			+					+				+	
25.1	Transcription	HVO_0819	D4GUE2	SirR/DtxR family transcription regulator SirR	+										+	+	+	
19.9	Transcription	HVO_1727	L9VGC6	TATA-box-binding protein	+			+					+		+			
20.3	Transcription	HVO_0158	D4GZA2	TATA-box-binding protein 1									+					
23.7	Transcription	HVO_2067	D4GU80	TetR family transcription regulator	+													
21.6	Transcription	HVO_A0161	L9V4Z6	TetR family transcriptional regulator	+													
19.8	Transcription	HVO_0163	L9UFW5	Transcriptional regulator	+		+	+				+	+		+	+	+	+
28	Transcription	HVO_0179	L9UGU5	Transcriptional regulator	+						+	+	+		+	+	+	+
27.5	Transcription	HVO_2928	L9UK32	Transcriptional regulator	+	+	+	+	+			+	+	+	+		+	+
39	Transcription	HVO_0568	L9UN99	Transcriptional regulator	+													
30.9	Transcription	HVO_2636	L9V878	Transcriptional regulator				+										
15.2	Transcription	HVO_1360	D4GXQ1	TrmB family transcription regulator	+													
39.3	Transcription	HVO_2688	D4GVQ2	TrmB family transcription regulator TrmB									+					
20.6	Transcription	HVO_1174	L9UT88	Transcription factor E	+								+					
35	Protein folding	HVO_0329	L9UK29	Branched-chain amino acid aminotransferase									+					
67.2	Protein folding	HVO_1590	L9VEI0	Chaperone protein DnaK	+										+			
15.2	Protein folding	HVO_0450	D4GRZ1	Hsp20-type molecular chaperone				+										
46.6	Protein folding	HVO_0321	D4GZV0	Peptide chain release factor subunit 1	+								+		+			
48.1	Replication and Repair	HVO_0853	D4GUK0	DNA double-strand break repair protein Mre11	+											+	+	
100	Replication and Repair	HVO_0854	D4GUK1	DNA double-strand break repair Rad50 ATPase	+										+	+	+	
78.7	Replication and Repair	HVO_0551	L9UMB1	DNA mismatch repair protein MutL	+								+					
99.2	Replication and Repair	HVO_0552	D4GSE9	DNA mismatch repair protein MutS	+								+		+	+	+	
37.3	Replication and Repair	HVO_0104	Q48328	DNA repair and recombination protein RadA	+			+				+	+		+	+	+	+
24.6	Replication and Repair	HVO_2383	L9V6G6	DNA repair and recombination protein RadB	+	+		+				+	+		+	+	+	+
76.8	Replication and Repair	HVO_1723	L9VE99	Putative DNA helicase Rad25	+								+		+	+	+	
94.8	Replication and Repair	HVO_1573	D4GZ02	DNA gyrase subunit A	+								+		+			
150.5	Replication and Repair	HVO_0858	L9US19	DNA polymerase B1	+								+					
26.7	Replication and Repair	HVO_0175	D0VWY8	DNA polymerase sliding clamp									+					
108.6	Replication and Repair	HVO_0393	L9UJI6	Excinuclease ABC subunit A									+					
76.3	Replication and Repair	HVO_0283	L9UK77	Helicase AshA									+					
42.4	Replication and Repair	HVO_1537	D4GYI1	Orc1-type DNA replication protein	+												+	
42.2	Replication and Repair	HVO_0194	L9UGW2	ORC1-type DNA replication protein	+							+	+		+		+	
24	Replication and Repair	HVO_3014	D4GYC9	Origin-associated GTP-binding protein	+								+					
69.6	Replication and Repair	HVO_0415	D4GRV6	Repair helicase UvrD				+					+					
34.6	Replication and Repair	HVO_0292	L9UJU8	Replication factor A				+					+					
53.4	Replication and Repair	HVO_2427	D4GSN1	Replication factor C large subunit									+					
36.2	Replication and Repair	HVO_0203	L9UG08	Replication factor C small subunit									+					
51	Replication and Repair	HVO_0519	D4GS55	Replication protein A									+					
41.8	Replication and Repair	HVO_1570	L9VEU6	Type 2 DNA topoisomerase 6 subunit A	+								+					
87.4	Replication and Repair	HVO_1571	L9VEG4	Type 2 DNA topoisomerase 6 subunit B				+					+		+			
75.4	RNA turnover	HVO_0783	L9UNA4	ATP-dependent protease Lon											+			
23.2	RNA turnover	HVO_2438	L9V6L9	Ribonuclease H I				+										
50.1	RNA turnover	HVO_2724	L9V5P6	Ribonuclease J	+	+				+			+					
25.6	RNA turnover	HVO_1094	D4GW15	Ribonuclease P protein component 3	+										+		+	
50.3	RNA turnover	HVO_0388	L9UJI9	Ribonuclease R	+							+	+		+	+	+	
34.1	RNA turnover	HVO_0144	L9UHJ9	Ribonuclease Z	+		+								+		+	+
67.4	Protein turnover	HVO_0829	D4GUG3	Peptidase S9 family protein	+								+				+	
27.6	Protein turnover	HVO_1091	Q9V2V6	Proteasome subunit alpha 1									+					
26.7	Protein turnover	HVO_2923	Q9V2V5	Proteasome subunit alpha 2									+					
26	Protein turnover	HVO_1562	L9VFN7	Proteasome subunit beta										+				
48.7	Protein turnover		L9UQ37	Proteasome-activating nucleotidase											+			
59.3	Protein turnover	HVO_0455	O30560	Thermosome subunit 2	+	+		+		+		+	+	+	+		+	
55.2	Protein turnover	HVO_0778	Q9HHA2	Thermosome subunit 3	+			+		+		+	+	+	+		+	+

Supplementary Table 7. The RNA polymerase subunits interaction network.
RpoD is shown in brackets as the yield after overproduction was very low.

				Bait						
MW [kDa]	Genloci	Accession	Description	rpoA1	rpoA2	rpoB1	rpoB2	(rpoD)	rpoH	rpoL
108.8	HVO_0349	D4GZX6	**rpoA1**	bait	+	+	+	+	+	+
46.1	HVO_0350	D4GZX7	**rpoA2**	+	bait	+	+	+	+	+
67.7	HVO_0348	L9UJM2	**rpoB1**	+	+	bait	+		+	+
58.9	HVO_0347	L9UK99	**rpoB2**	+	+	+	bait	+	+	+
28.1	HVO_2781	L9V5W2	**rpoD**	+	+	+	+	bait	+	+
8.5	HVO_0346	D4GZX3	**rpoH**	+	+	+	+		bait	+
10.4	HVO_1042	D4GVL8	**rpoL**	+	+	+	+		+	bait
7.3	HVO_2776	D4GWX5	**rpoN**	+	+	+	+		+	+
5.1	HVO_0653	L9UMK6	**rpoP**	+		+	+		+	+
13.6	HVO_2748	D4GWA3	**rpoF**						+	

Supplementary Table 8. Overview of aIFs co-isolated with RNA polymerase subunits.
RpoD is shown in brackets as the yield after overproduction was very low.

				Bait						
MW [kDa]	Genloci	Accession	Description	rpoA1	rpoA2	rpoB1	rpoB2	(rpoD)	rpoH	rpoL
10.8	HVO_1946	D4GTH5	**aIF1**							
11.5	HVO_0136	D4GZ79	**aIF1A-1**							
11.2	HVO_A0637	D4GRU5	**aIF1A-2**							
29.5	HVO_0699	D4GT46	**aIF2α**						+	
14.9	HVO_1678	D4GZP2	**aIF2β-1**							
22.2	HVO_2242	L9VAS4	**aIF2β-2**							
43.9	HVO_1901	D4GTD4	**aIF2γ**							
43.2	HVO_1934	D4GTG3	**aIF2Bsu**				+			
34.9	HVO_0966	L9USK7	**aIF2Bα**	+	+	+	+	+	+	+
30.7	HVO_2706	L9V7F9	**aIF2Bδ**							
104.5	HVO_1333	D4GXK1	**eIF4A-hom.**							
14.2	HVO_2300	L9V7A1	**aIF5A**							
65.3	HVO_1963	D4GTJ2	**aIF5B**	+	+	+	+		+	+
22.98	HVO_0117	D4GYW3	**aIF6**							

Supplementary Table 9. Overview of RNA polymerase subunits co-isolated with aIFs.

				Bait													
MW [kDa]	Genloci	Accession	Description	aIF1	aIF1A-1	aIF1A-2	aIF2α	aIF2-β1	aIF2-β2	aIF2-γ	aIF2Bsu	aIF2Bα	aIF2Bδ	eIF4A-hom	aIF5A	aIF5B	aIF6
108.8	HVO_0349	D4GZX6	**rpoA1**	+	+		+					+		+	+	+	
46.1	HVO_0350	D4GZX7	**rpoA2**	+			+					+		+			
67.7	HVO_0348	L9UJM2	**rpoB1**	+								+					
58.9	HVO_0347	L9UK99	**rpoB2**	+			+			+		+		+	+	+	
28.1	HVO_2781	L9V5W2	**rpoD**	+								+					
8.5	HVO_0346	D4GZX3	**rpoH**									+					
13.7	HVO_2748	D4GWA3	**rpoF**									+					
10.3	HVO_1042	D4GVL8	**rpoL**	+													

Supplementary Table 10. All (non-metabolic) proteins co-purified with RNA polymerase proteins.

Transcription factors that were also co-isolated with aIFs as baits are marked grey-blue.

				Bait						
MW [kDa]	Genloci	Accession	Description	rpoA1	rpoA2	rpoB1	rpoB2	(rpoD)	rpoH	rpoL
108.8	HVO_0349	D4GZX6	DNA-directed RNA polymerase subunit A1	+	+	+	+	+	+	+
46.1	HVO_0350	D4GZX7	DNA-directed RNA polymerase subunit A2	+	+	+	+	+	+	+
67.7	HVO_0348	L9UJM2	DNA-directed RNA polymerase subunit B1	+	+	+	+		+	+
58.9	HVO_0347	L9UK99	DNA-directed RNA polymerase subunit B2	+	+	+	+	+	+	+
28.1	HVO_2781	L9V5W2	DNA-directed RNA polymerase subunit D	+	+	+	+		+	+
8.5	HVO_0346	D4GZX3	DNA-directed RNA polymerase subunit H	+	+	+	+		+	+
10.4	HVO_1042	D4GVL8	DNA-directed RNA polymerase subunit L	+	+	+	+		+	+
7.3	HVO_2776	D4GWX5	DNA-directed RNA polymerase subunit N	+	+	+	+		+	+
5.1	HVO_0653	L9UMK6	DNA-directed RNA polymerase subunit P	+		+	+		+	+
13.6	HVO_2748	D4GWA3	DNA-directed RNA polymerase subunit F						+	
29.5	HVO_0699	D4GT46	Translation initiation factor 2 subunit alpha						+	
43.2	HVO_1934	D4GTG3	Translation initiation factor aIF-2B alpha subunit				+			
34.9	HVO_0966	L9USK7	Translation initiation factor IF-2B subunit delta	+	+	+	+	+	+	+
65.3	HVO_1963	D4GTJ2	Translation initiation factor aIF5B	+	+	+	+		+	+
28.3	HVO_2110	L9V9M0	ArcR family transcription regulator	+						
67.2	HVO_1590	L9VEI0	Chaperone protein DnaK					+		
17.7	HVO_0766	L9UNS7	Hsp20-type molecular chaperone	+						
12.6	HVO_1116	D4GW55	HTH domain protein			+		+		
11.7	HVO_1133	D4GW85	HTH domain protein	+		+	+	+	+	+
13.8	HVO_0962	D4GV69	HTH domain protein						+	
23.4	HVO_1695	D4H040	HTH domain protein	+		+	+		+	+
23.8	HVO_A0121	D4GQF3	HTH-10 family transcription regulator	+						
28	HVO_1501	D4GYE7	HTH-type transcriptional regulator GlpR	+					+	+
15.2	HVO_0450	D4GRZ1	Hsp20-type molecular chaperone						+	
28	HVO_2108	D4GUQ5	IclR family transcription regulator	+	+	+	+	+	+	+
27.9	HVO_A0583	D4GRP3	IclR family transcription regulator	+					+	
28.4	HVO_B0114	D4GPB5	IclR family transcription regulator	+	+	+	+		+	+
28.1	HVO_B0201	D4GPK2	IclR family transcription regulator	+			+	+		
18.1	HVO_1792	D4GSB6	Lrp/AsnC family transcription regulator	+		+	+	+	+	+
18.5	HVO_B0066	D4GP68	Lrp/AsnC family transcription regulator						+	
11.4	HVO_2869	D4GXH6	PadR family transcription regulator	+			+			+
25.1	HVO_0819	D4GUE2	SirR/DtxR family transcription regulator SirR	+		+	+		+	+
19.8	HVO_0163	L9UFW5	Transcriptional regulator	+		+	+	+	+	+
28	HVO_0179	L9UGU5	Transcriptional regulator	+		+	+		+	+
15.2	HVO_1360	D4GXQ1	TrmB family transcription regulator						+	
39	HVO_0568	L9UN99	Transcriptional regulator	+						
27.5	HVO_2928	L9UK32	Transcriptional regulator	+		+	+	+	+	+
30.9	HVO_2636	L9V878	Transcriptional regulator						+	
48.1	HVO_0853	D4GUK0	DNA double-strand break repair protein Mre11	+			+		+	
100	HVO_0854	D4GUK1	DNA double-strand break repair Rad50 ATPase	+		+	+	+	+	+
94.8	HVO_1573	D4GZ02	DNA gyrase subunit A						+	+
99.2	HVO_0552	D4GSE9	DNA mismatch repair protein MutS	+		+	+		+	+
150.5	HVO_0858	L9US19	DNA polymerase B1	+						
37.3	HVO_0104	Q48328	DNA repair and recombination protein RadA	+	+	+	+	+	+	+
24.6	HVO_2383	L9V6G6	DNA repair and recombination protein RadB	+	+	+	+	+	+	+
53.2	HVO_A0441	L9VPT6	DNA repair helicase Rad25	+						
76.8	HVO_1723	L9VE99	Putative DNA helicase Rad25	+		+	+	+		+

33.4	HVO_2558	D4GTZ0	30S ribosomal protein S3						+	
26.6	HVO_2552	L9V7Y2	30S ribosomal protein S4e	+	+	+	+		+	
23.3	HVO_2544	D4GTX6	30S ribosomal protein S5			+				
13.6	HVO_2373	D4GWM1	30S ribosomal protein S8e					+		
11.5	HVO_0360	L9ULL7	30S ribosomal protein S10	+		+			+	
18.9	HVO_2784	L9V5M6	30S ribosomal protein S13				+			
16	HVO_2560	L9V659	30S ribosomal protein S19P			+	+		+	
11.6	HVO_1896	D4GTC9	30S ribosomal protein S24e					+		
23.1	HVO_2757	P41199	50S ribosomal protein L1	+		+	+	+	+	+
25.6	HVO_2561	D4GTZ3	50S ribosomal protein L2	+		+	+	+	+	+
37.2	HVO_2564	L9V5K9	50S ribosomal protein L3	+		+	+	+	+	+
27	HVO_2563	D4GTZ5	50S ribosomal protein L4			+	+			
36.9	HVO_2756	P41198	50S ribosomal protein L10			+	+		+	
19.8	HVO_0484	D4GS24	50S ribosomal protein L10e			+				
16.8	HVO_2758	P41200	50S ribosomal protein L11	+		+	+	+		+
17.8	HVO_2542	L9V7W8	50S ribosomal protein L15			+				
22.5	HVO_0561	L9UPT6	50S ribosomal protein L15e	+		+	+		+	+
16.9	HVO_2559	L9V5K3	50S ribosomal protein L22	+	+	+	+		+	+
13.4	HVO_2553	L9V5Y5	50S ribosomal protein L24					+		
17.1	HVO_2543	L9V5X1	50S ribosomal protein L30	+		+	+		+	+
25.8	HVO_2547	D4GTX9	50S ribosomal protein L32e			+	+	+		
57.8	HVO_2575	L9V605	Elongation factor aEF-1 subunit alpha-like protein	+		+	+		+	+
45.6	HVO_2413	L9V6J4	Elongation factor 1-alpha	+	+	+	+		+	+
80.4	HVO_0356	L9UK07	Elongation factor 2	+	+	+	+	+	+	+
28.9	HVO_1284	D4GXB4	GTP cyclohydrolase III	+		+	+			+
33.5	HVO_2348	D4GWJ7	GTP cyclohydrolase MptA							+
67.4	HVO_0829	D4GUG3	Peptidase S9 family protein	+					+	
48.7		L9UQ37	Proteasome-activating nucleotidase						+	
46.6	HVO_0321	D4GZV0	Peptide chain release factor subunit 1	+		+			+	+
23.6	HVO_1931	D4GTG0	Probable GTP-binding protein EngB	+			+			+
8.3	HVO_2723	D4GW47	RNA-binding protein Lsm	+					+	
59.3	HVO_0455	O30560	Thermosome subunit 2	+	+	+	+	+	+	+
55.2	HVO_0778	Q9HHA2	Thermosome subunit 3	+		+	+	+	+	
14.3	HVO_3013	D4GYC7	Origin-associated protein						+	
42.2	HVO_0194	L9UGW2	ORC1-type DNA replication protein	+		+	+		+	+
42.4	HVO_1537	D4GYI1	Orc1-type DNA replication protein				+			
86.6	HVO_1703	D4H048	Putative DEAD/DEAH box helicase	+						
23.2	HVO_2438	L9V6L9	Ribonuclease H I	+			+	+	+	+
50.1	HVO_2724	L9V5P6	Ribonuclease J	+		+				+
25.6	HVO_1094	D4GW15	Ribonuclease P protein component 3	+			+	+	+	+
50.3	HVO_0388	L9UJI9	Ribonuclease R	+		+	+		+	+
34.1	HVO_0144	L9UHJ9	Ribonuclease Z	+		+	+		+	+
19.9	HVO_1727	L9VGC6	TATA-box-binding protein			+	+	+	+	
